# Systematic process evaluation of the conjugation of proteins to gold nanoparticles

**DOI:** 10.1016/j.heliyon.2021.e07392

**Published:** 2021-06-25

**Authors:** Pablo Fagúndez, Santiago Botasini, Juan Pablo Tosar, Eduardo Méndez

**Affiliations:** a Unidad de Bioquímica Analítica, Centro de Investigaciones Nucleares, Facultad de Ciencias, Universidad de la República, 11400, Montevideo, Uruguay; b Graduate Program in Chemistry, Facultad de Química, Universidad de la República, Uruguay; c Laboratorio de Biomateriales, Instituto de Química Biológica, Facultad de Ciencias, Universidad de la República, 11400, Montevideo, Uruguay

**Keywords:** Bioconjugation, Colloidal stability, Gold nanoparticles, Covalent attachment, Non-covalent attachment

## Abstract

The present work addresses some fundamental aspects in the preparation of protein-conjugated gold nanoparticles, in order to ensure an appropriate final product. Ten broadly available and/or easy to implement analytical tools were benchmarked and compared in their capacity to provide reliable and conclusive information for each step of the procedure. These techniques included transmission electron microscopy, UV/VIS spectroscopy, dynamic light scattering, zeta-potential, Fourier-transformed infrared spectroscopy, colloidal stability titration, end-point colloidal stability analysis, cyclic voltammetry, agarose gel electrophoresis and size-exclusion chromatography (SEC). Four different proteins widely used as adaptors or blocking agents were tested, together with 13 nm gold nanoparticles containing different surface chemistries. Among all tested techniques, some of the least popular among nanomaterial scientists probed to be the most informative, including colloidal stability, gel electrophoresis and SEC; the latter being also an efficient purification procedure. These three techniques provide low-cost, low time consuming, sensitive and robust ways to assess the success of the nanoparticle bioconjugation steps, especially when used in adequate combinations.

## 1 Introduction

Protein attachment to gold nanoparticles (AuNPs) is a very common procedure in diverse scientific fields [1, 2, 3] and is therefore no longer limited to research laboratories with experience in nanochemistry. Despite this being a desired outcome of decades of research in material sciences, the widespread use of protein-AuNP conjugates comes at a cost. Frequently, researchers are interested in the applications of AuNPs for analytical or clinical purposes and there are no current step-by-step guidelines for ensuring adequate characterization of the final reagent, hampering commercial or clinical applications of this technology [4]. The purpose of this work is to provide a set of characterization techniques that can be used to inform, evaluate, and validate each step in the process from nanoparticle synthesis to protein bioconjugation.

The general procedure for bioconjugation of proteins to AuNPs involves several steps, where the achievement of each stage, and the final application, depends on the success of all previous steps [5]. To achieve the goal of efficient protein binding to AuNPs, different strategies have been employed. These strategies can involve both covalent immobilization (typically by carbodiimide chemistry) and non-covalent adsorption [3, 6, 7, 8].

For analytical applications, bioconjugated AuNPs can be regarded as the specific analytical reagent for a given analyte. As such, their physicochemical characterization, purity and shelf life should be provided, as well as their behaviour as a blank in the analytical procedure. As a plasmonic nanomaterial, it is essential that specific properties, like the localized surface plasmon resonance (LSPR) band, are preserved, and that the adsorbed biomolecules should not be present in a free state competing for the same analyte [1, 3, 7, 9, 10]. Finally, but of paramount importance, if the bioconjugated nanoparticles are going to be used in a biological media, they should resist the aggregation triggered by the high ionic strengths of biological samples.

As for the use of gold nanoparticles in direct medical applications [11], the lack of suitable standards emerges as a handicap in the characterization of the bioconjugation procedure. The multiplicity of factors determining the physicochemical properties of the nanoparticles impose the need for precise control of the bioconjugation procedure, given that slight variations in the synthesis protocols can have a strong effect in the resultant product. Hence, multiple analytical methods and tools have been employed to fully characterize nanoparticulate systems providing varying degrees of information about the physicochemical state in a given solution [12, 13, 14, 15]. However, little has been discussed about the strengths and limitations of each of these techniques.

Herein, we discuss analytical methods and tools that provide relevant information on the success of each step in the bioconjugation of proteins to AuNPs. We analysed each step in the bioconjugation procedure, starting from the widely used 13 nm citrate-capped gold nanoparticles (AuNP-cit) [16] as substrates and including covalent and non-covalent biomolecule attachment procedures. We also tested four different representative proteins that are widely used in analytical setups as direct affinity binders (immunoglobulin G), protein adaptors (Streptavidin and Protein AG) or blocking reagents (Bovine Serum Albumin, BSA). We surveyed well-established techniques and optimized new low cost and easy-to-implement assays for the characterization of each step of the procedure, from the synthesis of gold nanoparticles to the isolation of protein-conjugated AuNPs. Therefore, we provide a comprehensive assessment of the information that can be retrieved from each technique, taken separately or in combination. This information is of paramount importance for reproducible research in bio-nanomaterials and the establishment of standardized procedures that can be applied broadly.

## 2 Materials and methods

### 2.1 Chemicals and solutions

All chemicals and solvents were of reagent grade and used without further purification. Chloroauric acid (HAuCl_4_.3H_2_O, 99.9 %, Sigma-Aldrich), sodium chloride (NaCl, 99 %, Anedra), potassium chloride (KCl, > 99 %, R. Benzo), potassium hexacyanoferrate (III) (K_3_Fe(CN)_6_, >99 %, Fluka), sodium nitrate (NaNO_3_, >99 %, Sigma-Aldrich) potassium bromide (KBr, spectroscopic grade, Pike Technologies), 11-mercapto-undecanoic acid (MUA, 95 %, Sigma-Aldrich), trisodium citrate dihydrate (99 %, Carlo Erba), sodium hydroxide (97 %, Anedra), N-hydroxysuccinimide (NHS, 98 %, Sigma-Aldrich), 1-ethyl-3-(3-dimethylaminopropyl) carbodiimide hydrochloride (EDC, 98 %, Sigma-Aldrich), sodium N-hydroxysulfosuccinimide (s-NHS, 98 %, Sigma-Aldrich), 2-(N-morpholino)ethanesulfonic acid (MES, 99 %, Sigma-Aldrich), sodium dihydrogen phosphate (NaH_2_PO_4_, >99 %, Sigma-Aldrich), disodium hydrogen phosphate (Na_2_HPO_4_, >99 %, Sigma-Aldrich), glacial acetic acid (HAc, 100 %, anhydro, Merck), sodium acetate trihydrate (NaAc, 99%, Merck), sodium bicarbonate (NaHCO_3_, >99 %, Sigma-Aldrich) bovine serum albumin (BSA, protease free, > 96 %, Capricorn Scientific), monoclonal anti-P15 antibody (Ab, ~150 kDa, 200 μg mL^−1^, IgG1 Santa Cruz Biotechnology), recombinant protein A/G (AG, ~50 kDa, 50 mg mL^−1^ Thermo Scientific), Streptavidine (Strp, ~55 kDa, Santa Cruz Biotechnology).

Strp was reconstituted in water in order to make a 1 mg mL^−1^ stock solution as indicated by the manufacturer, and further diluted in 100 mM sodium carbonate buffer pH 9.0. Ultrapure water (resistivity >18.2 MOhm cm) was obtained from a MilliQ water purification system and employed in the preparation of all solutions. Glassware was carefully cleaned by soaking in freshly prepared *aqua regia*, obtained by mixing concentrated HCl + HNO_3_ 3:1 v/v (*caution: aqua regia is extremely corrosive and should be handled under ventilated hood with protective personal elements*), washed repeatedly with ultrapure water, dried in an oven and stored protected from dust. Whatman Anotop syringe filters, 0.45 and 0.2 μm nominal pore sizes, were employed for colloidal solution clean-up.

### 2.2 Transmission electron microscopy (TEM)

Diluted AuNP-cit colloidal solutions were drop-cast (10 μL) onto a carbon-coated copper grid and air-dried at room temperature. TEM images were taken in a JEOL, model JEM 1010 transmission electron microscopy at an acceleration voltage of 80 kV. The diameter (*d*_TEM_) of more than 500 individual AuNPs were measured from 10 micrographs, employing FIJI software [17].

### 2.3 UV/VIS spectroscopy (UV/VIS)

UV/VIS extinction spectra were routinely measured in 1 cm-optical path disposable polystyrene cuvettes with a double beam spectrophotometer (Analytika Specord 200 Plus). For the extended spectral range below 350 nm, a 1 cm-quartz cuvette was employed. For most measurements, a spectral resolution of 0.5 nm was employed, except for the ligand exchange experiments, for which a value of 0.2 nm was employed (Supplementary Material SM1). The wavelength scan rate was 10 nm s^−1^.

### 2.4 Dynamic light scattering (DLS) measurements

The average hydrodynamic diameter (*d*_h_) and *ζ*-potential of the AuNPs were performed by means of dynamic light scattering (DLS) using a Brookhaven model ZetaPlus 90 equipped with a 659 nm laser, and a correlator for DLS at a fixed angle of 90° and 15° for DLS and *ζ*-potential measurements, respectively. DLS measurements, were taken in 1-cm optical path polystyrene cuvettes, following the ISO 22412 guidance [18]. For *ζ*-potential, an electrodic system (SZP-Surface Zeta Potential electrode, Brookhaven accessory) consisting in two parallel palladium electrodes was used. Measurements were conducted at 25 °C employing a 1 nM AuNP solution in 1 mM NaCl. (Supplementary Material SM2). Data (*n* = 6) were analyzed with the software Particle Solution v. 2.5 with the CONTIN and Smoluchowski algorithms for hydrodynamic diameter and *ζ*-potential, respectively.

### 2.5 Fourier-transformed infrared spectroscopy (FTIR)

FTIR spectra were obtained in the range 4000–400 cm^−1^ employing a Shimadzu infrared spectrometer model IR-Prestige 21, by averaging 10 scans at a nominal resolution of 4 cm^−1^, and Happ-Genzel apodization. For solid sodium citrate and MUA, samples were thoroughly mixed with KBr in an agate mortar, and 13 mm-discs were prepared in a Pike CrushIR at a pressure of 10 ton with the aid of a vacuum pump.

For the nanoparticle characterization, the colloidal solution is freed from excess capping agent by centrifugation (3X) at 10,000 *g* for 20 min at 4 °C and re-suspended in ultrapure water. The resulting solution was drop-cast onto a BaF_2_ window allowing for a complete drying between drops.

### 2.6 Colloidal stability titration

The assessment of the colloidal stability was determined automatically by adding increasing volumes (180 ± 30 μL) of NaNO_3_ 0.3 M at 2 min intervals with a peristaltic pump (FIA pump, Metrohm) to a 10 mL batch solution of gold nanoparticles under stirring conditions. The solution was continuously pumped into a 1-mL quartz flow cell inside the spectrophotometer. A microcontroller triggers the addition of NaNO_3_ solution at pre-set time intervals. Each 2 min, a UV/VIS spectral scan between 350 nm and 900 nm was registered and the absorbance maximum at *ca*. 520 nm (assigned to the presence of non-aggregated AuNPs) was corrected (to avoid the dilution effect), normalized and plotted against the increasing NaNO_3_ concentration of the colloidal dispersion.

### 2.7 Colloidal stability assay (end-point)

A simplified version of the colloidal stability titration assay was used as a quick screening method. It employed a 96-well microplate containing the modified AuNPs (100 μL) into which 100 μL of 300 mM NaCl was added. After thoroughly mixing, the 520/650 nm absorbance ratio was measured using a microplate reader (Tekan Infinite F50).

### 2.8 Electrochemical studies

Cyclic voltammetry was carried out at room temperature on a screen-printed electrochemical system (220AT, DropSens) consisting in a 4 mm diameter gold layer (geometric area 0.126 cm^2^) as working electrode, a truncated gold ring as counter electrode, and a silver quasi-reference electrode. The potential was controlled with a STAT400 DropSens potentiostat. Purified colloidal solutions were drop cast onto the working electrode and allowed to dry between each addition. Afterwards, 70 μL of a solution containing 1 mM hexacyanoferrate (III) in 0.1 M KCl were placed onto the whole electrodic system. After a preconditioning step at 0.50 V for 30 s, the potential was scanned at 0.050 V s^−1^ in the negative direction to -0.15 V, finishing the cycle in the positive direction to an upper potential value of 0.50 V. The peak potential difference, Δ*E*_p_, between the anodic and cathodic current peaks was annotated for the calculation of the heterogeneous rate constant, *k*_o_ [19].

### 2.9 Electrophoretic mobility assay

Electrophoretic mobility assays were conducted in an agarose gel (1 % w/v in TAE buffer, 40 mM Tris, 20 mM acetic acid, and 1 mM EDTA). 10–40 μL of the sample was seeded and the run was performed at 100 V for 5 min in an electrophoretic chamber (Cleaver Scientific, horizontal multiSUB-mini system). Relative migration distance (*R*_f_) was determined relative to AuNP-MUA.

### 2.10 Size exclusion chromatography (SEC)

To separate protein-modified AuNPs from free proteins and non-conjugated AuNPs, we employed a pre-packed size exclusion chromatography column (iZon Science, qEVoriginal, 70 nm nominal pore size, void volume = 3 mL). Briefly, the column was equilibrated with 50 mL of low ionic strength phosphate buffer (PB_low_: 5 mM, 5 mM NaCl, pH 7.4) and then 500 μL of the sample was seeded on top, forming a thin layer. Elution was carried out with the same PB_low_ buffer. Aliquots of 500 μL were collected up to a total elution volume (*V*_e_) of 20 mL, and transferred to a microplate. The absorbances at 280 nm (BSA) or 520 nm (free AuNPs) were measured using a double beam spectrophotometer (Analytika Specord 200 Plus) or microplate reader (Tekan Infinite F50), respectively. To assess the *V*_e_ for BSA, we employed 3 mg mL^−1^ BSA solution as a reference according to the manufacturer of the column.

### 2.11 Data treatment and simulations

Experimental data were analysed with Microcal Origin 2016. Mie simulations were carried out with MiePlot v.4.5.01 [20], assuming spherical 13-nm diameter particles, real and imaginary gold refractive index values of 0.150 and 3.601, respectively, 10 % size dispersion (maximum allowed) and 50 nanoparticles. Segelstein data was used to simulate water as surrounding media [21].

### 2.12 Synthetic procedures

#### 2.12.1 Synthesis of AuNP-cit

The Turkevich method [16] was followed based on the modifications introduced by Liu and Lu [22] and minor changes. A detailed mechanistic explanation for the formation of 13 nm AuNPs based on this method was recently published by our group [23]. Briefly, in a two-neck flask, 50 mL of ultrapure water and 50 μmol of HAuCl_4_.3H_2_O were added and heated to reflux under vigorous agitation and protected from light. Once the solution boils, 5 mL of 38.8 mM sodium citrate solution was quickly added. Solution colour turn from yellow to blue and finally red. Boiling and agitation are maintained for additional 15 min; afterwards, the solution is allowed to cool without agitation, transferred into a clean glass vial, and stored at 4 °C for further analysis.

#### 2.12.2 Synthesis of AuNP-MUA

Previous to the ligand exchange reaction, all glassware was soaked in 12 M NaOH aqueous solution for 1 h [22], thoroughly washed with ultrapure water and oven dried. One mL of the as-synthesized AuNP-cit (10 nM) was placed in a clean, NaOH-treated vial, and 20 μL of 500 mM MUA solution was added (AuNP-cit:MUA, 1:1000000 M ratio). The solution reaction was vigorously mixed and incubated at room temperature for 24 h. Afterwards, the solution was cleared by filtration through 0.45 μm to remove MUA aggregates and stored at 4 °C for further analysis.

### 2.13 Covalent immobilization of Ab on AuNP-MUA

Chemical crosslinking was carried out over AuNP-MUA using EDC/NHS chemistry [24]. The EDC/NHS activation was performed at low ionic strength phosphate buffer (PB_low_). For more information about buffer selection, see Supplementary Material SM3. Before surface activation with EDC/NHS, AuNP-MUA were centrifuged 3X at 10,000 *g* for 20 min at 4 °C and resuspended in PB_low_. Afterwards, 400 μL of AuNP-MUA (10 nM) was placed in a 1.5 mL-polypropylene tube, and 10 μL of EDC and 20 μL of NHS or s-NHS were simultaneously added and incubated for 5 min at room temperature. The EDC and NHS/s-NHS concentrations tested were 7.4, 3.7 and 1.85 mM and 14.8, 7.4 and 3.7 mM, respectively, keeping a fixed EDC:NHS/s-NHS 2:1 M ratio. Finally, 40 μL of 33 μg mL^−1^ Ab was added, and the mixture was incubated for 24 hs at 4 °C. The Ab concentration employed was maintained in high ionic strength PB_high_ (5 mM, 50 mM NaCl, pH 7.4).

### 2.14 Noncovalent adsorption of proteins on AuNP-MUA and AuNP-cit

First, optimal protein concentration and pH were obtained by incubating 10 μL Ab solutions at various concentrations (33, 66 and 132 μg mL^−1^ in water) with 100 μL of AuNP-cit or AuNP-MUA (10 nM) for 90 min in a 96 well microplate. The pH was adjusted before addition of the Ab, by addition of 5 μL of different 100 mM buffer solutions, namely, phosphate buffer (PB) in the pH range 5.8–7.4, borate buffer (BB) for 8.0 to 8.6, and carbonate buffer (CB) for 9.0 to 10.0. After incubation, the stability of the modified nanoparticles was measured by the end-point colloidal stability assay. Similar optimization was performed for BSA, Strp and protein AG.

**Figure 1:**
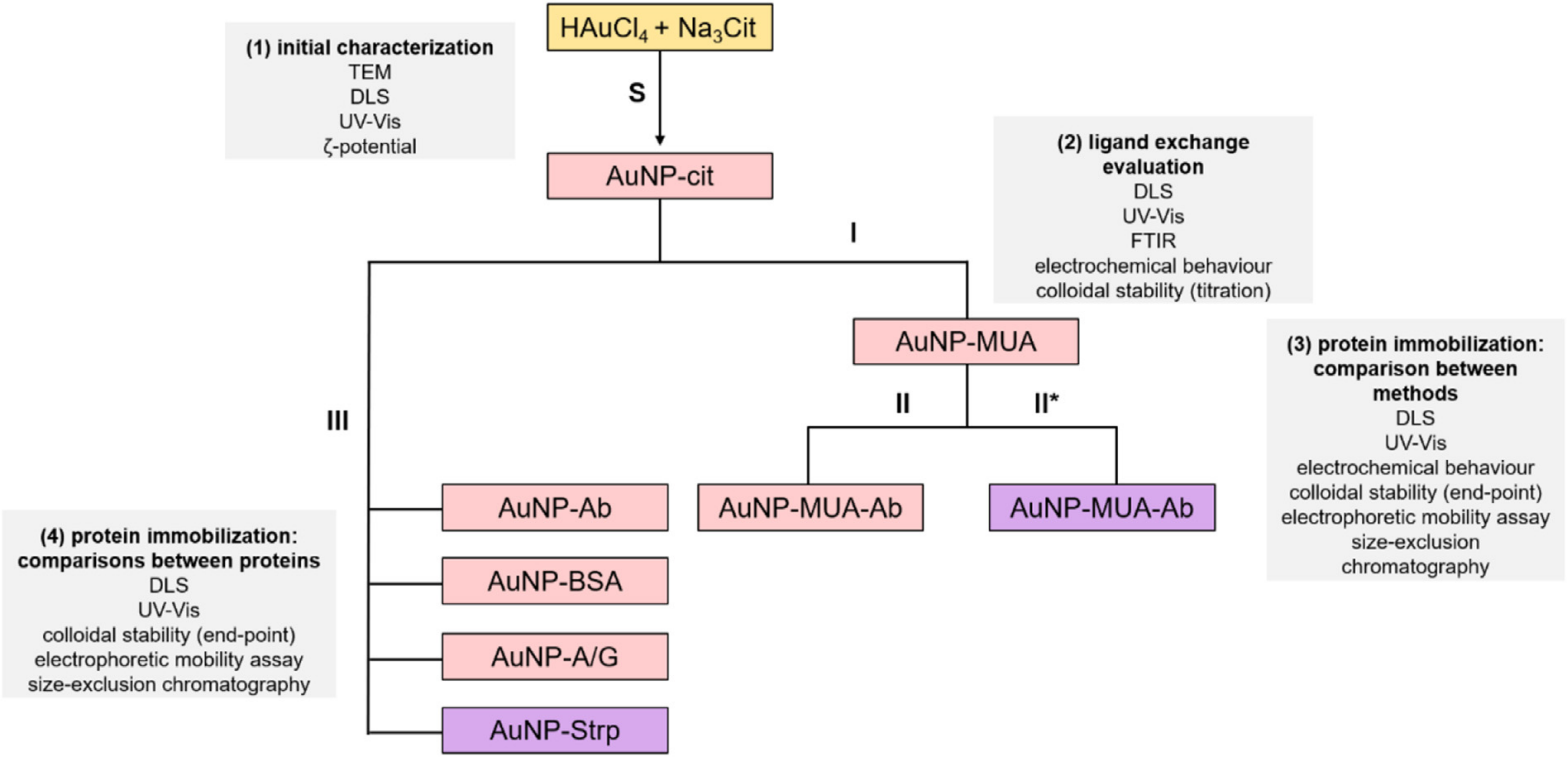
Overview of the study design and surveyed techniques. S: synthesis of AuNP-cit. I: Ligand exchange MUA/citrate. II: non-covalent Ab adsorption to AuNP-MUA. II∗: covalent Ab adsorption to AuNP-MUA. III: non-covalent protein adsorption to AuNP-cit. Grey boxes indicate the techniques used to evaluate the success of each step or to compare between variables (proteins or immobilization methods). Colours represent typical macroscopic characteristics of each solution/colloidal suspension.

Once optimal protein concentrations were defined, the bioconjugation reaction was scaled up by addition of 1000 μL of AuNP-cit (Ab, Strp, BSA, AG) or AuNP-MUA (Ab) to 100 μL of protein solutions. Protein-modified AuNPs were characterized by several techniques as indicated in Figure 1.

## 3 Results

We followed different approaches that are widely used in the scientific literature for the conjugation of proteins to AuNPs, starting from 13 nm AuNP-cit as a representative precursor [3, 6, 7, 8]. These approaches include either ligand exchange from citrate to a self-assembled monolayer of MUA (I) and covalent (II∗) or non-covalent (II and III) attachment of proteins to the AuNPs containing both ligands (Figure 1). Different physicochemical characterization techniques and separation assays were tested and critically evaluated for their capacity to inform success or failure of each step.

### 3.1 Synthesis and characterization of AuNP-cit

Strict adherence to the procedure details outlined in [22] yields a population of spherical gold nanoparticles with a mean diameter of 13 ± 4 nm (*n* = 510) (Supplementary Material SM4 Figure S5). The UV/VIS extinction spectra displays a LSPR band maximum at 522 nm, in accordance to reported results for 13-nm AuNP-cit (Supplementary Material SM4 Figure S6) [25, 26, 27]. The calculated molar extinction for AuNP-cit, was 2.3 × 10^8^ M^−1^ cm^−1^. Mie simulations for this size in water also predicts the maximum at 522 nm (Supplementary Material SM4 Figure S6), and indicate the predominance of absorption over scattering (Supplementary Material SM5). The *ζ*-potential of -36 ± 7 mV (*n* = 6) is in agreement with the ionic state of adsorbed citrate molecules at pH 5.4. DLS measurements, evaluated through the number of dispersing species and the intensity of the dispersed light, yield a main population with *d*_h_ of 16 ± 2 nm, along with a minor population of larger nanoparticles with mean *d*_h_ of 82 nm (Supplementary Material SM4 Figure S7).

Storage stability studies of AuNP-cit at 4 °C, show a slight increase in the absorbance maxima with storage time. DLS measurements indicate that some aggregates with *d*_h_ = 95 ± 5 nm are formed after 50 days, which represent *ca*. 400 of 13 nm-nanoparticles per individual aggregate. These clusters, clearly seen in DLS measurements, are barely noticeable by UV/VIS spectra, highlighting the superior capacity of DLS to detect early aggregation states (Supplementary Material SM6).

Finally, an absorbance value of 1.2 units was determined as the maximum value up to which the Beer – Lambert law is fulfilled, determining the limit for the use of the absorbance as an additive property [28] (Supplementary Material SM9 Figure S12).

### 3.2 Citrate to MUA ligand exchange

**Figure 2:**
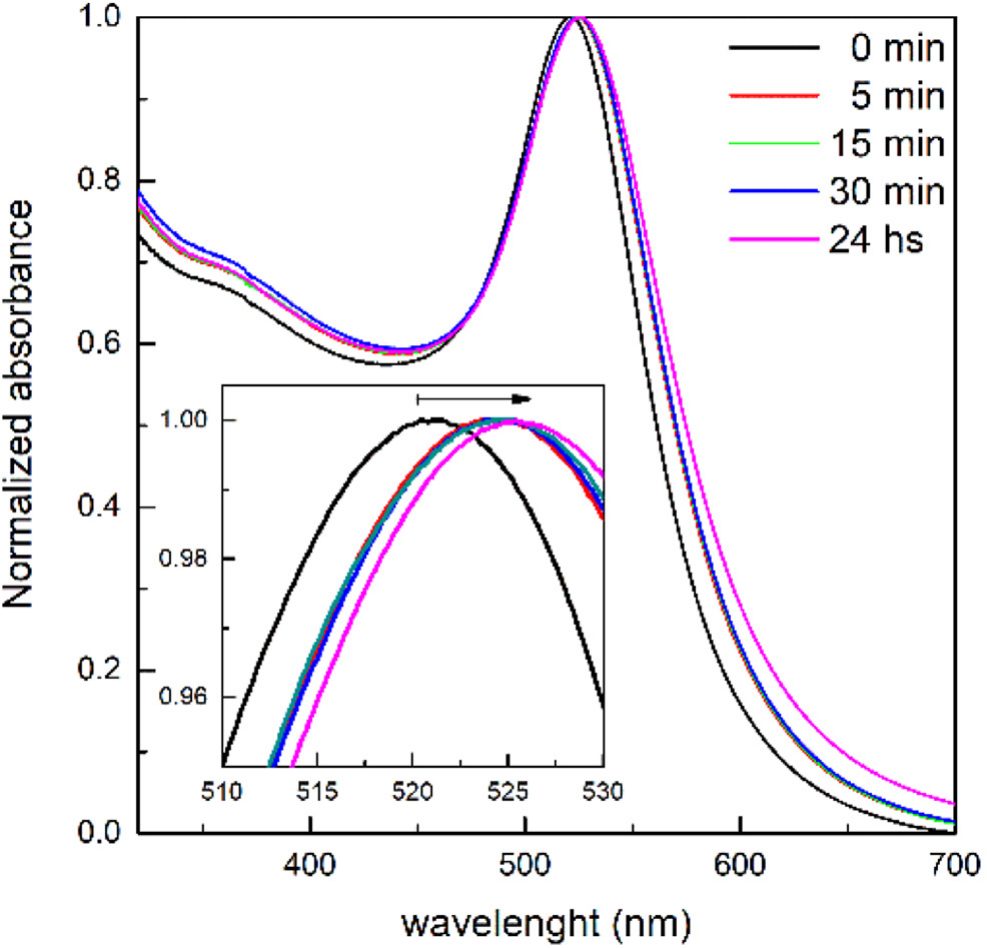
UV/VIS monitoring of AuNP-MUA formation during the ligand exchange reaction. Wavelength scan rate: 10 nm s^−1^, resolution: 0.2 nm.

Ligand exchange between MUA in solution and adsorbed citrate, followed for 24 h, produced a red shift in the LSPR band from 522 to 525 nm (Figure 2). The latter represents the final equilibrium value, after a rapid shift that could be appreciated in the first 30 min of reaction. The pH of the solution after the reaction remained around a value of 5.6, suggesting a ionized state of the surface exposed carboxylic group of adsorbed MUA [29], and in agreement with a measured *ζ*-potential of -23 ± 3 mV (*n* = 6). The average hydrodynamic diameters of 27 ± 2 nm (number) and 33 ± 2 nm (intensity) indicates that the modification procedure is consistent with the formation of MUA-capped nanoparticles with no appreciable aggregation (Supplementary Material SM7 Figure S10).

A simple method was envisaged to estimate the molar extinction coefficient of gold nanoparticles obtained by the ligand exchange method based on two assumptions: the ligand exchange between MUA and citrate is complete [30], and no loss of gold atoms takes place. The proposed method consists in the measurement of the absorbance maxima of serial dilutions of the original AuNP-cit and of the modified AuNP-MUA. The ratio between the Lambert-Beer slopes in the linear range equals the ratio between the respective molar absorption coefficients (Supplementary Material SM9 Figure S12). From the experimental ratio *ε*AuNP-cit/*ε*AuNP-MUA = 1.18, a value of 2.0 × 10^8^ M^−1^ cm^−1^ is estimated for *ε*AuNP-MUA.

FTIR analysis was used to evaluate the surface modification of AuNPs, since it is frequently reported as a method to assess ligand exchange procedures for these particular ligands. However, FTIR needs to be carefully implemented to distinguish between citrate and MUA, due to their similar spectral features. A detailed discussion on this topic can be found in Supplementary Material SM8.

**Figure 3:**
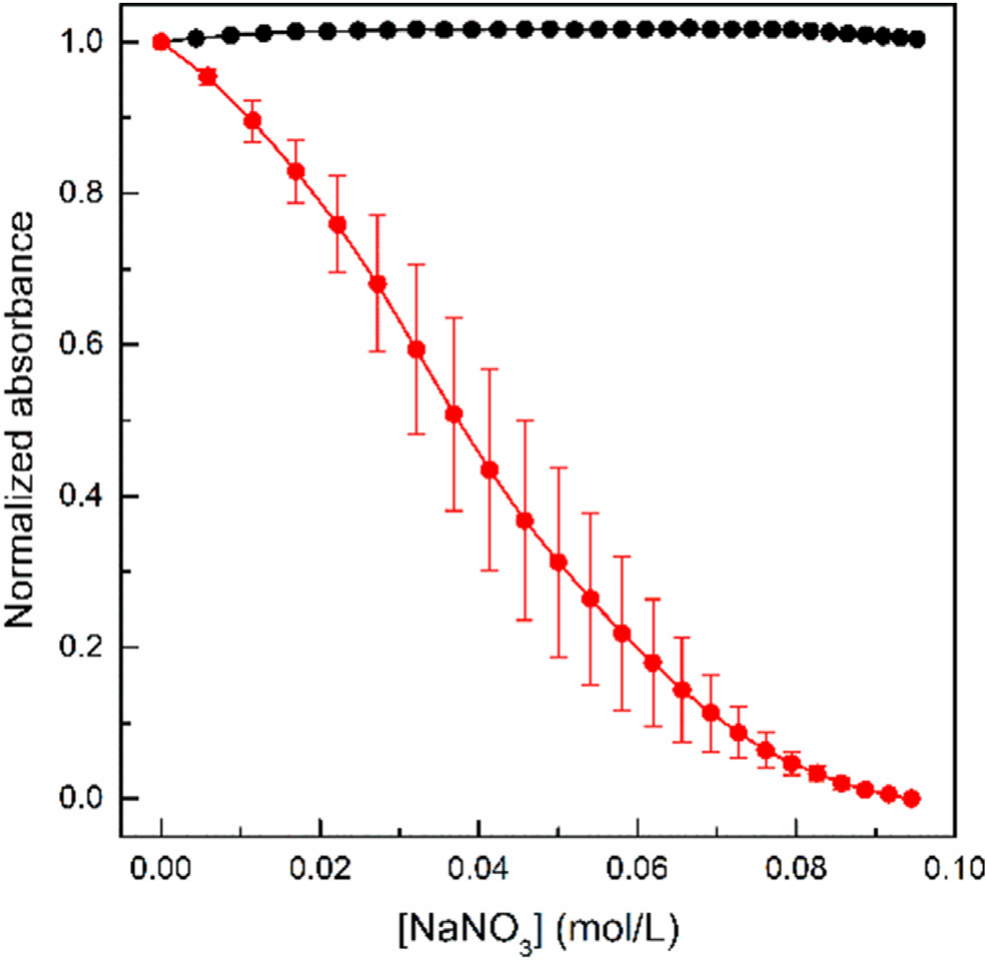
Aggregation curves of AuNP-cit (red) and AuNP-MUA (black) colloidal solutions with aqueous 0.30 M NaNO_3_. All data are normalized to a value of 1.0 for the Abs 520 nm at time = 0. Each curve represents the mean ± SD of three independent titrations.

In order to obtain an experimental assay that can yield conclusive results, we looked for alternative methods that could confirm the accomplishment of the ligand exchange. To do so, we implemented an automated system for the determination of the colloidal stability of the nanoparticles dispersion after progressive increases of the ionic strength of the medium with an inert salt, while monitoring the LSPR band in real time (Figure 3). A clear difference can be seen between AuNP-cit and AuNP-MUA, in agreement with the expected stabilization conferred by covalently bound MUA [31], thus providing compelling evidence for successful ligand exchange.

### 3.3 Covalent immobilization of antibodies by EDC/NHS chemistry

For the covalent crosslinking of Ab to AuNPs we employed AuNP-MUA and EDC in combination with NHS or s-NHS. First, excess MUA from AuNP-MUA colloidal dispersion was removed by centrifugation, and the AuNP-MUA re-suspended in PB_low_ buffer. It should be noted that the centrifugation and/or buffer exchange steps alone have a significant effect in AuNP-MUA stability. Indeed, at least some degree of aggregation took place, evidenced from the decrease in the 522 nm/650 nm absorbance ratio (Supplementary Material SM3).

After EDC/NHS activation and Ab incubation, we observed complete aggregation for the higher NHS concentrations assayed. For the lowest concentration of NHS, 3.7 mM, a red shift in the LSPR of 14 nm was observed, but could not be ascribed to the attachment of Ab, as the decrease in the 522/650 nm absorbance ratio revealed a partial aggregation state. The use of s-NHS instead of NHS yielded similar results, also observed by DLS analysis. At a s-NHS concentration of 3.7 mM, we obtained a red shift of 5 nm in the LSPR band position, with no net decrease in the 522/650 nm absorbance ratio (Supplementary Material SM10). However, this shift was also observed in the absence of Ab and DLS measurements showed the same increase in the *d*_h_ for s-NHS and NHS, so it was not possible to confirm the Ab bioconjugation based solely on these techniques (Supplementary Material SM11).

To solve this issue, we loaded the different colloidal suspensions in a 1 % agarose gel and performed electrophoresis to measure their migration towards the anode. The *R*_f_ values, determined relative to AuNP-MUA, were 0.95 and 0.71 for AuNP-MUA-s-NHS and AuNP-MUA-s–NHS–Ab, respectively, suggesting Ab immobilization. Despite this, a “smearing” pattern in the migration profile in both cases suggests that different particle sizes are present in the sample, indicating heterogeneity induced by s-NHS (data not shown).

### 3.4 Non-covalent adsorption of antibodies to AuNPs

**Figure 4:**
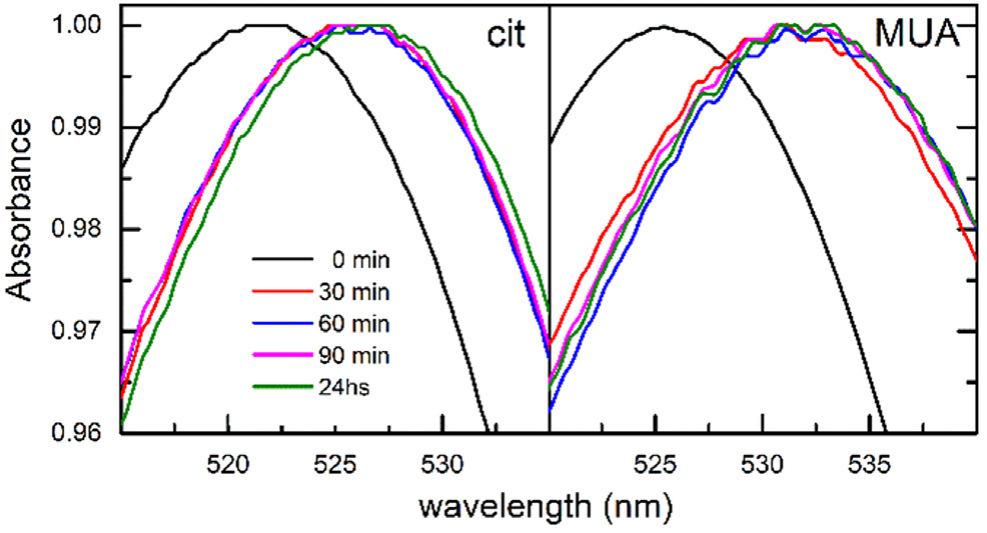
Time evolution of the LSPR band in UV/VIS spectra during Ab adsorption on AuNP-cit and AuNP-MUA.

Adsorption of Ab was followed by UV/VIS and DLS measurements. The red shifts in the LSPR band position from 522 to 525 nm (AuNP-cit) and from 525 to 530 nm (AuNP-MUA) were associated to the surface modification upon adsorption of Ab (Figure 4). These LSPR band shifts were observed as short as 30 min after incubation, and a similar behaviour was observed for *d*_h_ measured by DLS analysis (Supplementary Material SM12 Figure S14), which achieved a final value of 139 ± 3 nm and 255 ± 11 nm (by intensity) or 67 ± 22 and 92 ± 36 (by number), for AuNP-cit-Ab and AuNP-MUA-Ab, respectively. Considering that the reported *d*_h_ for an IgG is in the range 10–12 nm [32], we expected a hydrodynamic diameter around 41 or 51 nm for AuNP-cit-Ab and AuNP-MUA-Ab, respectively, considering the anchorage of two Ab molecules per AuNP in opposite positions. Thus, it is possible that DLS measurements are capturing the formation of aggregates involving a low number of AuNPs, but DLS measurements by number suggest that the overall aggregation state of the population is low, consistent with UV/VIS spectra (Supplementary Material SM12 Figure S15).

Beyond this, is important to mention that the protein adsorption onto AuNP surface can change the refraction index and dielectric constant of the surrounding medium. These effects can affect *d*_h_ values. Thus, the evaluation of the change in the hydrodynamic diameter, Δ*d*_h_, upon a modification event (Table 1) [33] is indicative of a modification but it cannot be directly compared to the theoretic diameter of a single IgG. In every step toward the final bioconjugated product, the *d*_h_ values significantly increased, in accordance with an expected increase in the size of the ensemble. Additionally, we suggest that the Δ*d*_h_ is a more appropriate indicator of the bioconjugation because the absolute number of dh could be different if some aggregates are present in the solution. Moreover, changes in pH or ionic strength can also modify the hydrodynamic diameter, and therefore the absolute value could change from one synthesis to another. However, for each synthesis, the Δ*d*_h_ cancels out the pH and the ionic strength effect, since the condition is the same. That is why this evidence should be accompanied with other complementary techniques.

**Table 1:** Change in the hydrodynamic diameter (Δ*d*_h_) for each step in the bioconjugation of Ab to AuNPs.

Process^a^	Δ*d*_h_ (nm) ± SD	
	by number	by intensity
I	10 ± 4	16 ± 4
II	65 ± 38	222 ± 13
III	51 ± 24	122 ± 5

a The bioconjugation steps correspond to those shown in Figure 1.

For electrophoretic mobility assays, in contrast to what we previously observed with AuNP-MUA-s–NHS–Ab, physically adsorbed Ab did not migrate (*R*_f_ = 0) in both AuNP-cit-Ab and AuNP-MUA-Ab (Supplementary Material SM13 Figure S16 and Figure S17). This result suggests that, in both cases, we have bigger and/or poorly charged particles that are not able to migrate out of the sample wells. For unmodified AuNP-cit nanoparticles, the high ionic strength of the electrophoretic buffer caused aggregation at the application point (evidenced by the sample turning blue immediately upon loading in the gel) but not for AuNP-cit-Ab. Thus, agarose gel electrophoresis offered compelling evidence suggesting successful Ab bioconjugation in both AuNPs.

The *ζ*-potential for AuNP-cit-Ab and AuNP-MUA-Ab were -8 ± 6 mV and -10 ± 8 mV at pH 5.2, respectively, implying a change in the surface-ionisable groups of AuNPs after Ab adsorption (see above). This change toward neutrality is consistent with the observed absence of electrophoretic mobility in conjugated nanoparticles. The effect of Ab concentration and pH in the adsorption process on AuNP-cit was negligible, as deduced from the constancy in the 522/650 nm absorbance ratio at 140 mM NaCl. However, for AuNP-MUA-Ab, a dependency on Ab concentration and bioconjugation reaction pH was observed (Supplementary Material SM14), possibly indicating electrostatic repulsion between the Ab and negatively-charged MUA when the pH was set above the isoelectric point of the antibody (see a detailed discussion in Supplementary Material SM14).

### 3.5 Electrochemical evaluation of different AuNP-cit modification steps

Another way to corroborate the changes in the nanoparticle surface is to adsorb the different nanoparticles onto gold electrode surfaces and observe how their presence affects the capability of the electrodic system to transfer electrons to the hexacyanoferrate (III) species in solution (Supplementary Material SM15). Such capability can be assessed through the experimental determination of the Δ*E*_p_ (Supplementary Material SM15 Table S3), which is related to the heterogeneous rate of charge transfer, *k*_o_ [34].

For all electrode modifications, both anodic and cathodic currents associated to hexacyanoferrate (III)/(II) redox system are observed indicating that the charge transfer process is not completely blocked. The deposition of AuNP-cit is reflected in a slight increase in the measured current, in agreement with a larger electroactive area produced by the adsorption of AuNPs, and with a marginal decrease in the value of *k*_o_. Interestingly, Ab-modified AuNPs showed an increase in Δ*E*_p_ and a sharp decrease in *k*_o_, indicating that cyclic voltammetry can be used as an informative technique to evaluate the bioconjugation event. In all cases, the experimentally determined parameter, Δ*E*_p_, can be easily determined with an associated error below 5 %.

### 3.6 Stability of protein-AuNP composites is strongly protein-dependent

Next, we tested whether different proteins could be conjugated to AuNP-cit by the noncovalent adsorption method, given that it yielded the best results for the immobilization of antibodies. Three additional proteins were chosen based on their versatility and frequency of use in the scientific literature. Protein A/G (AG) and Streptavidin (Strp) were included since they are widely use as adaptors for the orientated immobilization of antibodies and biotin-modified proteins, respectively [35]. Bovine serum albumin (BSA) was also tested since it is frequently used as a blocking agent.

**Figure 5:**
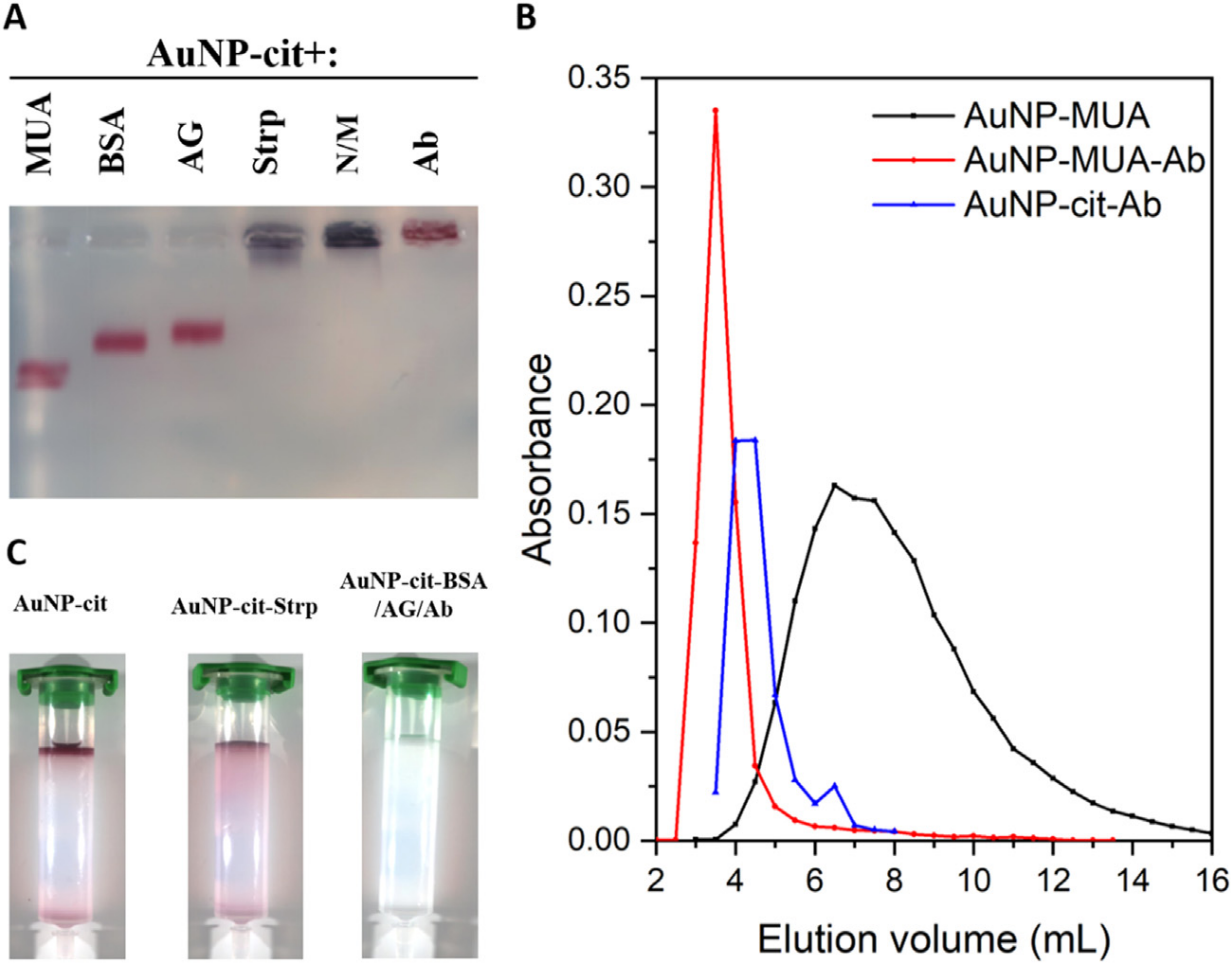
Purification and evaluation of noncovalent attachment of proteins to AuNP-cit by electrophoresis (A) and SEC (B). Images of SEC columns after injection of AuNP-cit, AuNP-Strp or subsequent injections of AuNP-cit-BSA, AuNP-cit-AG and AuNP-cit-Ab are provided (C). N/M: not modified.

The optimal concentration of each protein was first determined by colloidal stability assays (Supplementary Material SM16 Figure S20). Interestingly, while salt-induced aggregation of AuNP-cit-Strp was observed irrespectively of the concentration of the ligand (up to 200 μg/mL), Ab, AG and BSA yielded stable nanoparticles from concentrations as low as 80 μg/mL onwards. By UV/VIS analysis, Ab, AG and BSA showed the characteristic 3 nm red shift of the LSPR band, while Strp induced nanoparticle aggregation (Supplementary Material SM16 Figure S21). This was evident by a rapid change on the colour of the suspension and a tendency of the resultant nanoparticles to sediment after relatively low periods of time (>1 h vs days for unmodified AuNP-cit). It should be noted that this effect could be triggered by either Strp alone or undisclosed chemical agents included in the commercial Strp solution that was used. Similar results were obtained by DLS (Supplementary Material SM16 Table S4) and gel electrophoresis (Figure 5A and Supplementary Material SM16 Figure S22).

### 3.7 Characterization and purification of protein-AuNP composites by SEC

A final step in the preparation of protein-modified AuNPs is the purification of the modified nanoparticles from free soluble proteins that could interfere with analytical application of these reagents. This is usually performed by centrifugation [36]. However, we have consistently observed partial aggregation of the resultant nanoparticles after centrifugation and resuspension (data not shown). To achieve efficient nanoparticle purification without this drawback, we implemented a size-exclusion chromatography (SEC) method that is fast, reproducible, easy to use and does not require any specialized equipment. Additionally, this method can also be used to characterize and validate the bioconjugation reaction, as shown below.

The chromatograms (Abs 520 nm) of AuNP-MUA after injection to commercial pre-packed qEV columns (Izon, void volume = 3 mL) showed a broad peak at a *V*_e_ of 7 mL (Figure 5B). Interestingly, injection of AuNP-MUA-Ab resulted in a much narrower peak at *V*_e_ = 3.5 mL (Δ*V*_e_ = 3.5 mL, Figure 5B). Furthermore, this technique shows that antibody modification of the nanoparticles is virtually complete since no signal corresponding to unmodified AuNP-MUA was detected.

In contrast, AuNP-cit did not enter the columns and were retained in the pre-column filter (Figure 5C). Interestingly, the chromatograms of AuNP-cit-Ab (Figure 5B), AuNP-cit-BSA, or AuNP-cit-AG (Supplementary Material SM16 Figure S23) showed a log normal-like distribution peaking at *V*_e_ = 4.2 (Ab) or *V*_e_ = 6 mL (AG and BSA). This can be considered as further proof of protein immobilization because: a) the protein-modified AuNP-cit did enter the columns and b) they were also larger in size than unmodified AuNP-MUA (Δ*V*_e_ ≥ 1 mL), consistent with DLS results. Furthermore, soluble BSA showed a *V*_e_ = 8.5 mL, with negligible amounts contaminating the fractions where most AuNP-cit-BSA eluted (Supplementary Material SM16 Figure S23 panel B), demonstrating that SEC is a suitable method for purification of protein-modified nanoparticles. Like AuNP-cit, AuNP-cit-Strp nanoparticles were mostly retained in the prefilter of the columns, which turned violet rather than red in this case (Figure 5C). However, in contrast to AuNP-cit, a fraction of the population could enter in the column and a small peak with *V*_e_ = 4 mL was observed (recovery <10% based on the area under the curve vs AuNP-cit-BSA).

Taking these and previous results into consideration, we conclude that Streptavidin is being incorporated into the nanoparticles, yielding modified AuNPs that have a strong tendency to aggregate into large and heterogeneous ensembles; only a small fraction of them being able to cross the prefilters and enter the qEV columns. In contrast, BSA, AG or Ab conjugates were highly stable, even under high ionic strengths conditions (Supplementary Material SM16 Figure S20) and could be stored at room temperature for at least one week without changes in the 520/650 nm absorbance ratio (data not shown).

## 4 Discussion

The Turkevich procedure for citrate-capped gold nanoparticles is fully employed in nanoscience and nanotechnology [37], though the exact mechanism is still subject to continuous revisions [23, 38, 39, 40]. The synthetic procedure employed gives AuNP-cit of 13 nm if carefully done [22].

According to our results, the characterization of the synthesized AuNP-cit needs the complete set of experimental measurements, namely, UV/VIS, DLS, and TEM to fully describe the nanoparticulate system. Mie simulations and geometrical considerations provide a mean for the calculation of the molar absorption coefficient, but only considering the metallic core of the nanoparticles and do not reflect the physicochemical surroundings, which also affects their scattering ability. Mie simulations provide a suitable approach to determine the size of AuNPs in colloidal solutions, independently of the nature of the capping agent.

In the case of the molar extinction coefficients, data reported for AuNPs-cit are sufficiently consistent to be used with confidence. Our proposed procedure to assess the molar extinction coefficient for other capping agent is based on the assumption that ligand exchange is 100 % efficient. Hence, molar extinction coefficients for AuNPs colloidal solutions can be calculated from serial dilutions of the original AuNP-cit and the ligand exchanged AuNP, to determine the Lambert-Beer slopes.

Regarding the stability, DLS measurements constitute an efficient tool towards the detection of the early stages of nanoparticle aggregation [41]. In the first 15 days of storage, the mean value of *d*_h_ from DLS data was 40 ± 3 nm (by intensity) (Supplementary Material SM6). Considering a 95 % confidence interval, particles above 50 nm in diameter should be taken as the minimum detectable aggregates by DLS technique, corresponding to a representative aggregate composed by *ca*. 57 nanoparticles of 13 nm TEM diameter. This 10 % increase in the scattering efficiency should be reflected in an equivalent increase in the measured absorbance, a prediction indeed observed in the time-evolution spectra of the AuNP-cit stock solution. Notice that AuNPs that remain close between each other, not representing an actual increase in the absorption cross section, should compose these initial aggregates. At the same time, the LSPR band coupling is still not strong enough to be detected as an absorbance increase around 600 nm [42, 43]. Consequently, both UV/VIS spectra and DLS measurements are adequate to follow AuNP aggregation, while DLS measurements can be confidently used to assess the presence of AuNP aggregates in the initial stage.

The exchange reaction of citrate ligands by MUA is supported on several assumptions. First, as MUA is adsorbed on the AuNP through a strong covalent Au–S bond, simple thermodynamic considerations favours such reaction [44]. The reaction is assumed to be quantitative, that is, no citrate moiety remains attached, and a MUA monolayer is formed [30, 45]. Despite its relative simplicity, methods to assess the actual accomplishment of the reaction may be questionable. A typical red-shift between 2 – 5 nm of the LSPR band (depending on the capping agent) is usually taken as an indirect proof that the exchange reaction took place, and indeed this shift is quite robust and supported by several reports [3, 7, 46]. Beyond such robustness, the detection of this minute shift in the position of the LSPR band maximum requires that the spectral resolution of the spectrophotometer be set appropriately. Most spectrophotometers use a default value of 1 nm for the spectral resolution, and a band shift between 2 – 5 nm would imply an error between 100 – 40 % (Supplementary Material SM1). Hence, to use the LSPR band shift as a proof for the ligand exchange reaction, the spectral band resolution should be set as low as possible. In our case, we set that value in 0.2 nm, and the LSPR band shift of 3 nm for the MUA ligand exchange could be estimated with a 13 % error. Only under these experimental considerations, LSPR band shift turns into a robust result to assess the ligand exchange reaction.

The electrostatic stability results can be interpreted in terms of the influence of the ionic strength in the colloidal stability [47]. Comparative curves for AuNP-cit and Au-MUA colloidal solutions clearly show a different stability upon increase in the ionic strength of the nanoparticle surroundings.

The main difference between both nanoparticulate systems is that in the case of AuNP-cit, the capping agent is electrostatically adsorbed, while MUA is covalently attached to the AuNP surface. Hence, the potential screening effect in AuNP-cit is more effective towards the destabilization of the colloidal system. As a quantitative output of this analysis, the concentration of salt that produce a decrease of 50 % of the maximum absorbance value is termed as “critical concentration”, *C*_crit_. In the case of a stable system like AuNP-MUA, no *C*_crit_ can be estimated, and for AuNP-cit we obtained a value of *C*_crit_ = 37 mM NaNO_3_. The different behaviour between both systems is outstanding, and we can conclude that the study of the electrostatic stability of the colloidal systems provides a very conclusive experimental method to assess the ligand exchange reactions between citrate and thiols.

Covalent bioconjugation of proteins to AuNP-MUA involves many steps and each of them could affect the final product stability. Therefore, we suggest more than one technique for product evaluation. For example, the LSPR band red shift in the UV/VIS spectra suggests Ab attachment by EDC/NHS-mediated crosslinking, but a deeper analysis employing DLS and electrophoretic mobility showed EDC/NHS-induced aggregation, even when substituting NHS for s-NHS.

When we evaluated the noncovalent adsorption procedure, we obtained similar red shifts in the LSPR band position after 60 min of incubation, suggesting Ab attachment. In this case, the spectra were more defined and narrower. However, in the light of our previous results, we analysed AuNPs by DLS measurements and electrophoresis. In the case of DLS, for both AuNP-cit-Ab and AuNP-MUA-Ab, we observed an expected increase in the *d*_h_ during incubation, but the polydispersity index was lower compared to covalent attachment. We consider that these changes are due to the products of bioconjugation and not to an aggregation process, although DLS measurements by intensity did suggest the presence of a few larger particles.

In contrast to our electrophoretic mobility results for covalent bioconjugation, the Ab noncovalent adsorption products showed a significant *R*_f_ decrease without a smearing profile, suggesting a more effective Ab adsorption for both, AuNP-cit-Ab and AuNP-MUA-Ab. At the light of these results, we suggest that electrophoretic mobility is a useful technique to confirm the bioconjugation event.

The effect of the Ab concentration and pH during incubation was evaluated by a modification of our colloidal stability titration. The successful attachment of Ab was confirmed based on a comparison of the 520/650nm absorbance ratio after an increase of the ionic strength by addition of NaCl. High ratios were obtained when employing AuNP-cit for all Ab concentrations and pH assayed. This suggests a protective effect of the protein corona after an increase in ionic strength, also confirming Ab attachment.

The pH independence in the case of the physical adsorption to 13 nm AuNP-cit could indicate that the Ab interact directly with the gold surface, displacing citrate molecules. In contrast, in the case of adsorption to AuNP-MUA, Ab are expected to interact with MUA carboxylic acid groups without displacing covalently attached MUA. In this case, the ionic state of these carboxylic groups affects the possibility of Ab approximation, with a sharp drop in bioconjugation efficiency observed for pH > 8. Above this pH, we expect both MUA and Ab to be negatively charged, and this would hinder their approach. Beyond this interpretation, the colloidal stability assay is a rapid and useful tool to evaluate different experimental conditions and confirm the immobilization of antibodies.

Electrochemical evaluation of the heterogeneous charge transfer rate to a soluble redox probe also yielded highly conclusive data. For example, the modification of the gold electrode with AuNP-cit produces a decrease in the *k*_o_ values of 19 %, determined from the increase in the Δ*E*_p_ values in 8 mV, an easily detectable quantity by voltammetry. Further adsorption of Ab on AuNP-cit-Ab increases the Δ*E*_p_ value in 76 mV. Hence, electrochemical evaluation of the nanoparticle-modified gold electrodes provides a very conclusive proof of the different steps in the bioconjugation process by noncovalent adsorption to AuNP-cit.

The nanoparticles modified by adsorption of Ab were also evaluated by size exclusion chromatography. The chromatograms obtained for AuNP-cit-Ab and AuNP-MUA-Ab showed lower *V*_e_ values for both products and a very narrow distribution, compared to unmodified AuNP-MUA (AuNP-cit did not enter the columns). These changes are consistent with an increase in the AuNP size due to Ab adsorption and confirm that Ab remains attached to the AuNPs even after removal of excess free antibodies. Additionally, SEC allowed us to discriminate between different protein-modified AuNP-cit. Thus, SEC can be employed not only as a purification technique, but also as a qualitative tool to confirm the bioconjugation process.

For the whole bioconjugation procedure, we have finally arrived at a proposal of appropriate analytical tools for the verification of the accomplishment of each step (Table 2).

**Table 2:** Quality of the information provided by different techniques (and parameters evaluated) in each of the steps involved in the bioconjugation of AuNPs.^a^

Technique	Purpose of the evaluation (parameter)^a^^,^^b^			
	1	2	3	4
TEM	++++ (*d*)	- (dns)	ND	ND
UV/VIS	++++ (*λ*_LSPR_)	++ (Δ*λ*_LSPR_)	++ (Δ*λ*_LSPR_)	++ (520/650 nm)
DLS	++++ (*d*_h_, PI)	+++ (Δ*d*_h_, PI)	++ (Δ*d*_h_, PI)	-
*ζ*-potential	++ (*ζ*)	++ (Δ*ζ*)	+ (Δ*ζ*)	ND
FTIR	++ (υ)	-	ND	ND
Colloidal stability (titration)	++ (*C*_crit_)	++++ (Δ*C*_crit_)	AuNP-cit: ++++ AuNP-MUA: -(Δ*C*_crit_)	ND
Colloidal stability (end-point)	-	++++ (520/650 nm)	AuNP-cit: ++++ AuNP-MUA: -(520/650 nm)	++ (520/650 nm)
Electrochemistry	-	++++ (dns) (Δ*E*p, *k*_0_)	AuNP-cit: ++++ (Δ*E*p, *k*_0_)	ND
Electrophoresis	-	++++ (Δ*R*_f_)	AuNP-cit: -AuNP-MUA: ++++ (Δ*R*_f_)	++ (Δ*R*_f_)
SEC	-	++++ (*V*_e_)	AuNP-cit: ++++ AuNP-MUA: ++++ (V_e_, Δ*V*_e_)	++++ (V_e_, Δ*V*_e_)

a Based on the nomenclature used in Figure 1. 1) Characterization of AuNP-cit. 2) ligand exchange (cit → MUA) evaluation. 3) Ab immobilization on AuNP-cit and AuNP-MUA. 4) Comparison between proteins immobilized on AuNP-cit.

b From “+” (poor/ambiguous) to “++++” (conclusive), “-”: not relevant information. “ND”: experiment not done. “dns”: data not shown.

## 5 Conclusions

A combination of techniques needs to be taken together to obtain a correct interpretation of the physical phenomena involved in each step of the bioconjugation process. However, some techniques are sufficiently informative for a conclusive proof of the accomplishment of the different steps, while others can be misleading if considered alone. We have provided a critical evaluation of ten different techniques and their utility to inform about different stages of the process.

Techniques such as gel electrophoresis and SEC are widely used in biochemistry and molecular biology and gave us highly conclusive results for all main bioconjugation steps. However, they are not widely used in nanomaterials laboratories despite being relatively fast, simple, and not requiring expensive equipment. In the case of SEC, it also allows for an efficient separation of bioconjugated nanoparticles from their non-conjugated counterparts and free proteins remaining in solution. Thus, we recommend these techniques to be included as part of the essential toolbox for bioconjugation process control.

In summary, this work constitutes an important step forward towards the standardization of step-by-step control procedures for nanomaterials such as protein-AuNP conjugates. We expect this effort will increase reproducibility in this field, aiding both producers and final users of nanoparticle-based analytical assays.

## Declarations

### Author contribution statement

Pablo Fagúndez: Conceived and designed the experiments; Performed the experiments; Analyzed and interpreted the data; Wrote the paper.

Santiago Botasini: Conceived and designed the experiments; Performed the experiments; Analyzed and interpreted the data.

Juan Pablo Tosar, Eduardo Méndez: Conceived and designed the experiments; Analyzed and interpreted the data; Contributed reagents, materials, analysis tools or data; Wrote the paper.

### Funding statement

This work was supported by 10.13039/501100006049Comisión Sectorial de Investigación Científica (CSIC, Universidad de la República, Uruguay) through Young Researchers Project C-164-348, and 10.13039/100008725Agencia Nacional de Investigación e Innovación (ANII, Ministerio de Educación y Cultura, Uruguay) for the doctoral fellowship ANII POS_NAC_2016_1_130350 awarded to PF. SB, JPT and EM are ANII and PEDECIBA researchers.

### Data availability statement

Data included in article/supplementary material/referenced in article.

### Declaration of interests statement

The authors declare no conflict of interest.

### Additional information

No additional information is available for this paper.
