# Supplementary material for: Systematic process evaluation of the conjugation of proteins to gold nanoparticles
Source: Heliyon. 2021 Jun 25;7(6):e07392. doi: 10.1016/j.heliyon.2021.e07392 (PMC8258641; doi:10.1016/j.heliyon.2021.e07392)
Supplement: Supplementary Material [file mmc1.docx]

**Systematic process evaluation of the conjugation of proteins to gold nanoparticles**

Supplementary Material (SM)

Pablo Fagúndez,^a^ Santiago Botasini,^b^ Juan Pablo Tosar,^a^ and Eduardo Méndez.*^b^

^a^ Unidad de Bioquímica Analítica, Centro de Investigaciones Nucleares, Facultad de Ciencias, Universidad de la República, 11400 Montevideo, Uruguay.

^b^ Laboratorio de Biomateriales, Instituto de Química Biológica, Facultad de Ciencias, Universidad de la República, 11400 Montevideo, Uruguay.

*Email: emendez@fcien.edu.uy, Tel/Fax: +598-25250749

Content

[SM1. Optimization of UV/VIS measurements 2](#_Toc74825143)

[SM2. Optimization of DLS measurements 4](#_Toc74825144)

[SM3. Buffer selection for the covalent attachment of Ab on AuNP-MUA 5](#_Toc74825145)

[SM4. Characterization of AuNP-cit 6](#_Toc74825146)

[SM5. Mie simulations 10](#_Toc74825147)

[SM6. Storage stability of AuNP-cit at 4 °C 11](#_Toc74825148)

[SM7. Hydrodynamic diameter for synthetized AuNP-MUA through ligand exchange 12](#_Toc74825149)

[SM8. FTIR analyses 13](#_Toc74825150)

[SM9. Molar extinction coefficient calculation for AuNP-MUA 15](#_Toc74825151)

[SM10. UV/VIS spectra after chemical conjugation of Ab to AuNP-MUA 16](#_Toc74825152)

[SM11. Hydrodynamic diameters after chemical conjugation of Ab to AuNP-MUA 17](#_Toc74825153)

[SM12. Time evolution of UV/VIS spectra and hydrodynamic diameter for the physical adsorption of Ab on AuNP-cit y AuNP-MUA 18](#_Toc74825154)

[SM13. Electrophoretic mobility assay for AuNP-cit and AuNP-MUA after Ab adsorption. 19](#_Toc74825155)

[SM14. The effect of Ab concentration and pH in the adsorption 21](#_Toc74825156)

[SM15. Electrochemical evaluation 22](#_Toc74825157)

[SM16. Characterization of different AuNP-cit-protein preparations 24](#_Toc74825158)

[References 28](#_Toc74825159)

## SM1. Optimization of UV/VIS measurements

The selection of the spectral resolution instrumental parameter in the UV/VIS spectrophotometer is key to obtain reproducible absorption spectra. In particular, when the precise assessment of the LSPR band maxima is needed, for example, as a probe of the success in the ligand exchange reaction, the red-shift is usually below 5 nm.

In order to optimize the adequate spectral resolution of our measurements, we performed the measurements summarized in Figure S1, repeating the spectral scan for a fixed spectral resolution in the range 0.2 – 1 nm, at different scan rates in the range 5 – 50 nm/s, which covered integration times of the measuring point between 0.1 and 0.002 s.





**Figure S1. Influence of the instrumental parameters “spectral resolution” and “spectral scan rate” in the acquisition of the UV/VIS spectra of AuNP-cit.** The insets provide a closer view to the curves at the LSRP band maxima.

As expected, the lower the spectral resolution, the higher the reproducibility of the acquired spectra. The LSPR band maxima show no significant differences between spectral resolutions of 0.2 nm and 0.5 nm. A spectral resolution of 1 nm, obviously, do not detect differences in the LSPR band maxima (Table S1).

According to these results, all spectra in this work were taken with a spectral resolution of 0.5 nm, and to assess the success in the ligand exchange reaction, we used a value of 0.2 nm. In all cases, the selected scan rate was 10 nm/s, which represent a good compromise between reproducibility and speed of analysis.

**Table S1. LSPR band properties as a function of the spectral resolution set in the spectrophotometer.**

| Spectral resolution (nm) | LSPR_max_ range (nm) | LSPR_max_ (nm) |
| --- | --- | --- |
| 0.2 | 521.8 – 522.8 | 522.3 ± 0.4 |
| 0.5 | 522.0 – 522.5 | 522.5 ± 0.5 |
| 1 | 523 | 523 |

## SM2. Optimization of DLS measurements

Optimization of hydrodynamic diameter and *ζ* -potential from DLS measurements is carried out in supported media, in order to provide enough electrical conduction in solution by ions. However, the presence of ions could trigger nanoparticle aggregation, and it is necessary to optimize the best concentration of NaCl to carry out the measurement, we tested NaCl concentrations from 0 to 10 mM (Figure S2).

Intensity

Number

**Figure S2. DLS measurements for AuNP-cit.** A: results obtained by evaluation of the intensity of the dispersed light. B: results obtained by evaluation of the number of the dispersing species. In both evaluations, the NaCl concentration employed varied between 0 and 10 mM.

As deduced from the DLS measurements by number of dispersing species, there is no significant difference in the concentration range 1 – 10 mM. However, measurement by intensity of the light dispersed in the absence of a supporting media (no NaCl) clearly indicates that a minimum ionic strength should be present in order to obtain a lower dispersion of sizes.

## SM3. Buffer selection for the covalent attachment of Ab on AuNP-MUA

The EDC/NHS activation procedure was performed at low ionic strength buffers. Several buffers were considered, to finally select PB_low_ as the most adequate.

In all cases, previous to the chemical activation procedure, AuNP-MUA were centrifuged 3X at 10,000 *g* for 20 min at 4 °C and re-suspended in the buffer. The centrifugation step also produces some degree of aggregation in the colloidal solution (Figure S3)


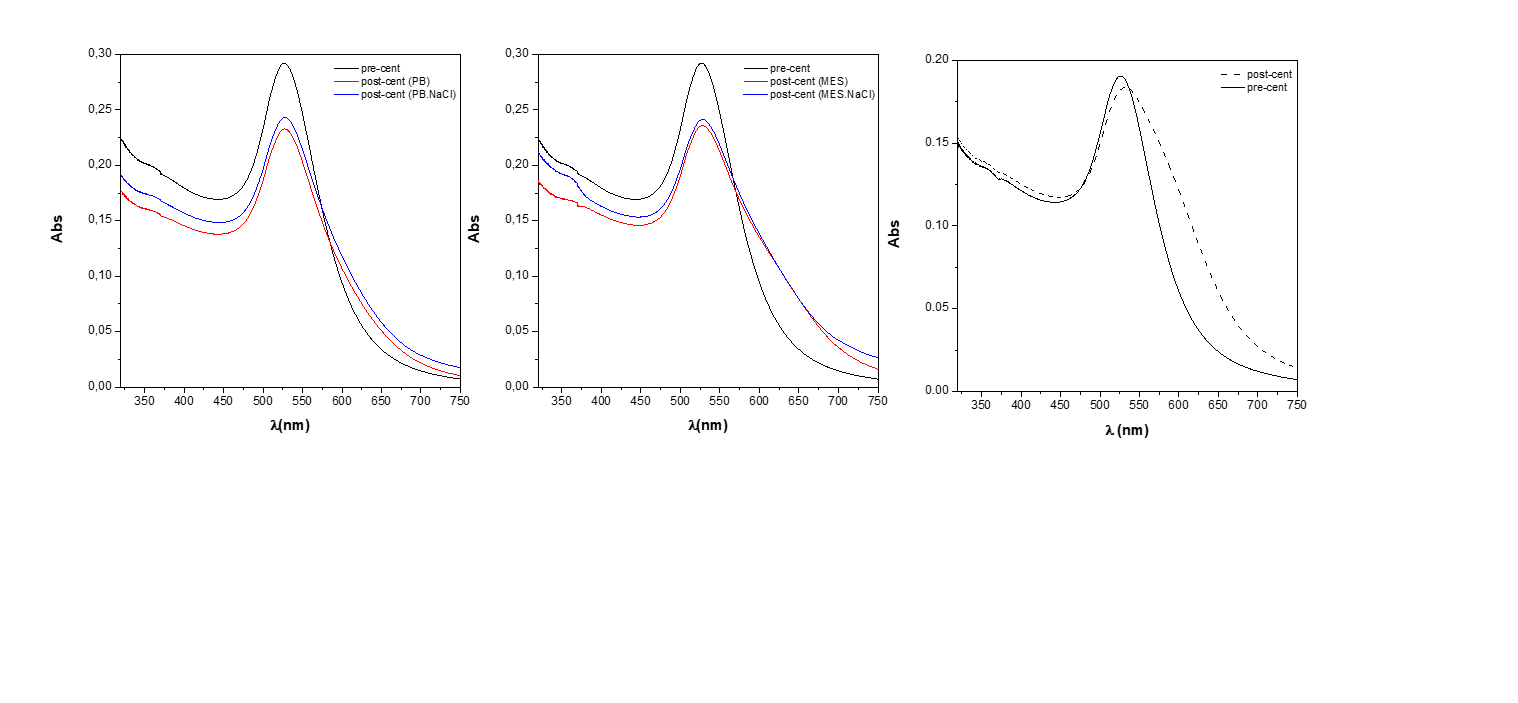


**C**

**B**

**A**

**Figure S3. UV/VIS spectra after 3 centrifugation/resuspension cycles.** A: resuspension in phosphate buffer (PB, 5 mM, 5 mM NaCl, pH = 7.4); B: resuspension in 2-(N-morpholino)ethanesulfonic acid (MES, 5 mM, 5 mM NaCl, pH = 5.0); C: comparative spectra before and after centrifugation and resuspension in the same AuNP-MUA reaction mixture.

The DLS measurements showing the effect of centrifugation and buffer employed in the resuspension are shown in Figure S4.

A B C D

**Figure S4. DLS measurement by intensity of the dispersed light for A: Before the centrifugation process, B: after 3 centrifugation/resuspension cycles, C: re-suspended in MES buffer, and D: re-suspended in PB buffer. In all cases, DLS measurements were carried out in NaCl 1 mM solution.**

## SM4. Characterization of AuNP-cit

Strict adherence to the procedure details outlined by Liu and Lu [[1](#_ENREF_1)] yields a population of spherical gold nanoparticles with a mean diameter of 13 ± 4 nm (*n* = 510) (Fig. S5). Gaussian fit to the population histogram yields a mean diameter of the fit of 12.9 ± 0.1 nm, with the nanoparticle population extending between 8.4 and 17.5 nm (95 % confidence limits).

To probe the degree of isolation/aggregation of the obtained AuNP-cit, we explored the following techniques: UV/VIS spectroscopy, Mie simulations, *ζ*-potential and DLS.


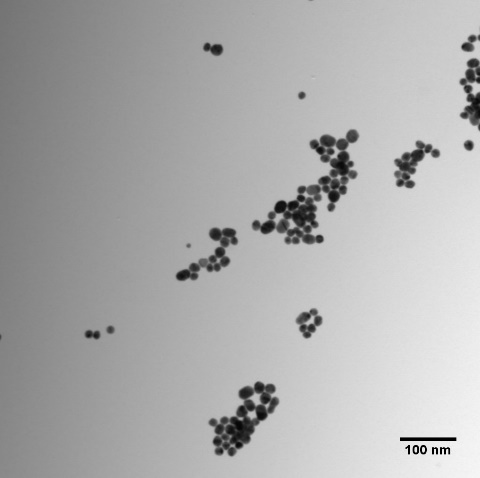

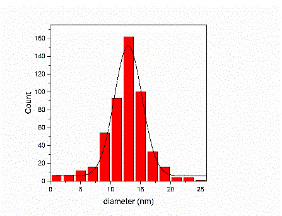


**Figure S5. TEM image of AuNP-cit.** Inset: Size distribution of the measured nanoparticles (*n* = 510) and Gaussian fit. Magnification: 10,000X.

UV/VIS spectroscopy and Mie simulations

The UV/VIS extinction spectra displays a LSRP band maximum at 522 nm (Fig. S6), in accordance to reported results for 13-nm AuNP-cit [[2-4](#_ENREF_2)]. The molar extinction for AuNP-cit was calculated via different considerations. On a pure geometrical basis, considering a complete reduction of the gold salt and nanoparticle density equal to the bulk (19.32 g cm^-3^), the calculated value is 2.1 x 10^8^ M^-1^ cm^-1^. Calculations based on interpolation of existing data yield comparable results: 2.4 x 10^8^ M^-1^ cm^-1^ [[2](#_ENREF_2)], 2.2 x 10^8^ M^-1^ cm^-1^ [[5](#_ENREF_5)], and 2.5 x 10^8^ M^-1^ cm^-1^ [[6](#_ENREF_6)]. Hence, the mean value representative for the molar extinction coefficient of 13 nm AuNP-cit is 2.3 x 10^8^ M^-1^ cm^-1^.

Mie simulations for this size in water also predicts the maximum at 522 nm, and the calculated spectral shape is symmetrically centered within the experimental one when a nanoparticle size dispersion of 10 % (maximum value allowed by the software) is considered (Fig S6). This observation agrees with an actual size dispersion of ca. 30 %, and that the synthesized AuNP-cit are not aggregated, as it should show an asymmetric spectral broadening biased to higher wavelengths. The experimental full width at half maximum (FWHM) of the LSPR band is 51 nm, a rather large value [[7](#_ENREF_7)], in agreement with the size dispersion obtained by TEM.

Mie simulations also indicate that the spectral extinction is mostly due to absorption rather than scattering (Supplementary Material SM5), and the estimated scattering efficiency, defined as the fraction of the total light scattered, is almost negligible [[5](#_ENREF_5)].





**Figure S6. Normalized experimental and simulated spectra of the AuNP-cit colloidal solution.**

*ζ*-potential and DLS measurements

The experimental pH value of the synthesis reaction, 5.4, dictates that citrate molecules in solution are mainly doubly deprotonated. The *ζ*-potential value of -36 ± 7 mV (*n* = 6) is in agreement with this.

DLS measurements were analysed evaluating the number of dispersing species and the intensity of the dispersed light (Fig S7). Despite the intensity distribution showed to populations with mean *d*_h_ of 17 nm and 82 nm, the larger aggregates only constitute a minor population in the colloidal solution, consistent with TEM results. In agreement with this, when performing DLS analysis by number instead of intensity, a single population of 16 ± 2 nm was observed.

The polydispersity index is relatively high, 0.273, which is in agreement with the FWHM of the UV/VIS spectral band. The slightly larger value of the hydrodynamic diameter compared to the TEM measurements is expected, since the latter only considers the metallic nucleus instead of the electrochemical double layer.





**Figure S7. DLS measurements for AuNP-cit by number and intensity, at 1 mM NaCl.** Full lines represent log-normal fitting to the data distribution.

Accomplishment of Beer – Lambert law

The maximum absorbance value up to which the Beer – Lambert law is fulfilled is determined by the linear range of the plot absorbance-concentration. If the synthesis reaction is complete, and taking into account the mean size of the AuNP-cit nanoparticles determined by TEM, it is possible to calculate the concentration of the colloidal solution and determine the upper value of the linear range. For our nanoparticle size, such absorbance value is ca. 1.2 absorbance units. All measurements involving the use of the absorbance as an additive property (kinetic and equilibrium studies, sensors, etc.) must be within the lower and upper limits where Beer-Lambert Law is valid [[8](#_ENREF_8)].

## SM5. Mie simulations

The Mie simulations of monodispersed AuNPs were carried out using MiePlot v.4.5.01, assuming spherical nanoparticles in the size range 8 – 60 nm, real and imaginary gold refractive index values of 0.150 and 3.601, respectively, 50 nanoparticles, and. Segelstein data to simulate water as surrounding media.


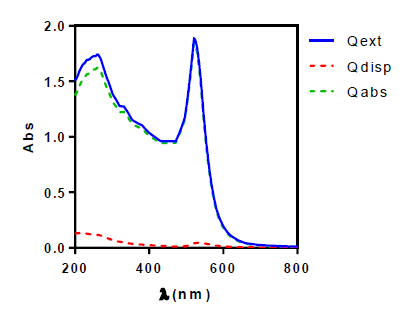




A

B

**Figure S8. A: Spectral simulations of AuNP extinction as a function of size, B: Absorption (Qabs) and Scattering (Qdisp) contributions to the extinction spectra (Qext) for AuNP 13 nm size.**

## SM6. **Storage stability of AuNP-cit at 4 °C**

AuNP-cit stored at 4 °C for 50 days, show a slight increase in the absorbance maxima with storage time. Normalization of all spectra show no changes in the general shape, suggesting that no significantly aggregation took place during storage as no broadening of the spectral band was observed. However, DLS measurements indicates that after 50 days some aggregates are indeed formed with an equivalent *d*_h_ = 95 ± 5 nm, which represent *ca*. 400 of 13 nm-nanoparticles per individual aggregate. These clusters are clearly seen in DLS measurements, but are only revealed in the slight increase in the UV/VIS spectra, reflecting an increase in the dispersion efficiency of the colloidal solution.

A

B

**Figure S9. A: Normalized UV/VIS spectra for AuNP-cit during storage at 4 °C for 48 days.** B: the same samples analyzed through variation in hydrodynamic diameter.

## SM7. Hydrodynamic diameter for synthetized AuNP-MUA through ligand exchange

B

A

**Figure S10. DLS measurement for AUNP-MUA, A: by intensity of dispersed particles, B: by number of dispersing particles.**

## SM8. FTIR analyses

FTIR analysis can be used for the evaluation of AuNP surface modifications [[9](#_ENREF_9)]. We compared the FTIR spectra of AuNP-cit and AuNP-MUA obtained on a BaF_2_ window. In relation to the parent compounds, we expected signals for the carboxylate group bound to the AuNP surface for citrate [[10](#_ENREF_10)], and unbound for MUA, as the latter anchors to the gold surface via the thiol group [[11](#_ENREF_11)]. However, as not all carboxylate groups are attached to the surface, also unbound carboxylate is expected for AuNP-cit. In addition, the CH_2_ group vibrations associated to the large scheletum of MUA are also expected for citrate molecule, though in a minor contribution. The interpretation of the vibrational bands for AuNP-cit is still debated [[11-14](#_ENREF_11)]. Our results showed new features for FTIR signals for AuNP-cit compared to the parent compound (Fig. S11). The original absorption band assigned to the stretching vibration of carboxylate group centred at 1591 cm^-1^ is replaced by two absorption bands centered at 1568 and 1552 cm^-1^, corresponding to the surface coordination of protonated citrates [[11](#_ENREF_11), [13](#_ENREF_13)], the relevant citrate species at the working pH from where the sample was taken.





**Figure S11. Normalized FTIR spectra for AuNP-MUA and AuNP-cit (black lines) and their respective pure parent compounds (red lines).**

Compared to the parent organic compound, adsorbed MUA through the SH moiety expose an ionized carboxylic group, through the absorption bands at 1567 cm^-1^ (asymmetric) and 1413 cm^-1^ (symmetric), which is in accordance to the ionization to carboxylate due to the solution pH from which the sample was taken, above its p*K*_a_ of 4.0 [[15](#_ENREF_15)]. Other absorption bands could indicate the good quality of the MUA self-assemblage onto the AuNP, like bending of methylene group at 1466 cm^-1^ in free MUA, is absent in the nanoparticles [[16](#_ENREF_16)]. In addition, the strong C-H stretching vibrations at 2918 and 2847 cm^-1^ in pure MUA significantly decrease the intensity, and shift to 2920 cm^-1^ and 2850 cm^-1^, in agreement to the formation of well-ordered domains of adsorbed MUA. These shifts have been reported both in 2D and 3D gold surfaces [[17](#_ENREF_17), [18](#_ENREF_18)]. Notice that these conclusions are based on an extremely detailed analysis of minor bands, which difficult the analysis.

Summing up, FTIR analysis provides several features that should be carefully analyzed in order to obtain results relevant to the adsorption state of each molecule. Comparison of AuNP-cit and AuNP-MUA show minute differences that should be analyzed very carefully. As the general expected features are the same for all modifications analyzed (CH_2_, carboxylate), FTIR does not provide strong experimental evidence on the modification of the nanoparticles surface, in particular, to the achievement of the ligand exchange reactions. Hence, FTIR analysis could be used as a tool to demonstrate the effectiveness of ligand exchange, depending of course on the IR features of the capping agents involved, which may require an extremely detailed analysis.

## SM9. Molar extinction coefficient calculation for AuNP-MUA

The molar extinction coefficient of gold nanoparticles depends on the nanoparticle size and shape, but also on the capping agent [[6](#_ENREF_6), [19](#_ENREF_19)]. Several works provide the size dependence of the molar extinction coefficient for gold nanoparticles capped with citrate ions [[2](#_ENREF_2), [3](#_ENREF_3), [6](#_ENREF_6), [19](#_ENREF_19)]. However, for other capping agents, such data are scarce, and usually data from AuNP-cit are used as a default value for all AuNPs [[20](#_ENREF_20)].

$\frac{\text{ε}\text{AuNP/Cit }\text{ }}{\text{ε}\text{AuNP/}\boldsymbol{MUA}}$=1.18

**Figure S12. Calibration curve for AuNP-MUA and AuNP-cit.** Spectra were carried out in water in a polypropylene cell (optical path 1 cm) and the absorbance at 522 nm was graphed. The results showed the mean of three absorbance values for each concentration.

## SM10. UV/VIS spectra after chemical conjugation of Ab to AuNP-MUA


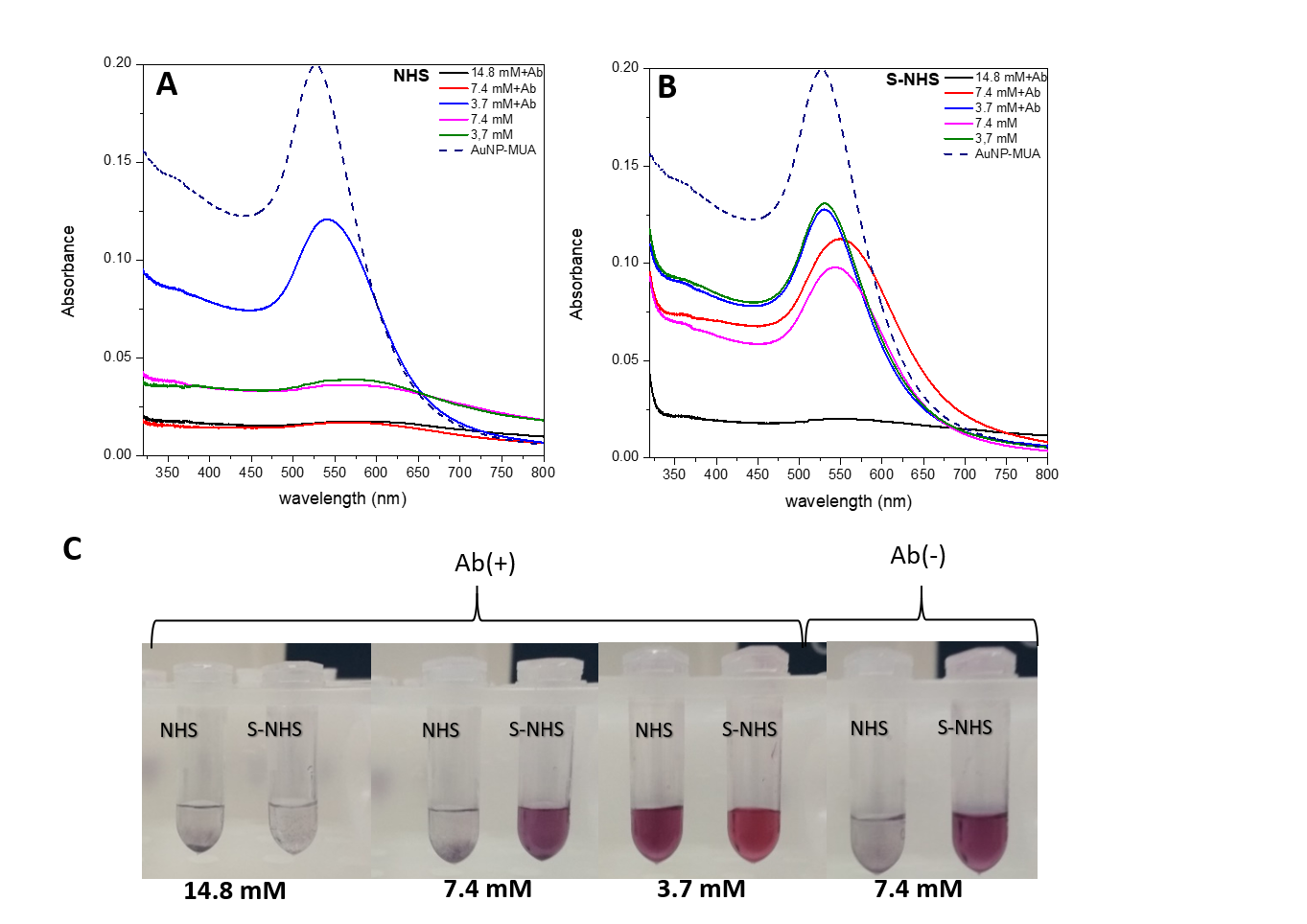


**Figure S13. UV/VIS analyses after the chemical bioconjugation procedure.** A: chemical bioconjugation with EDC/NHS, B: chemical bioconjugation with EDC/s-NHS, C: visual results of the colloidal solutions obtained.

## SM11. Hydrodynamic diameters after chemical conjugation of Ab to AuNP-MUA

**Table S2. Hydrodynamic diameters by DLS and polydispersion index (*PI*) after chemical bioconjugation with EDC/s-NHS in PBS in NaCl 1 mM.**

| [Ab] (µg/mL) | [s-NHS]_f_ mM | *d*_eff_ (nm) | *d*_h_ (nm) intensity | *d*_h_ (nm) number | PI |
| --- | --- | --- | --- | --- | --- |
| 33 | 14.8 | > 1,000 | 109 ± 17  > 1,000 | 79 ± 8 | 0.52 |
| 33 | 7.4 | 207 ± 4 | 66 ± 9  301 ± 20 | 61 ± 4 | 0.30 |
| 33 | 3.7 | 133 ± 2 | 50 ± 13  221 ± 26 | 46 ± 11 | 0.25 |
| 0 | 7.4 | 119 ± 2 | 49 ± 11  178 ± 17 | 44 ± 12 | 0.22 |
| 0 | 3.7 | 128 ± 2 | 43 ± 6  203 ± 31 | 40 ± 5 | 0.27 |

## SM12. Time evolution of UV/VIS spectra and hydrodynamic diameter for the physical adsorption of Ab on AuNP-cit y AuNP-MUA





**Figure S14. Change in the hydrodynamic diameter for AuNP-cit and AuNP-MUA during Ab adsorption.**

AuNP-cit

AuNP-MUA

A

B

**Figure S15. UV/VIS spectra for AuNP-cit (A) and AuNP-MUA (B) during Ab adsorption.**

## SM13. Electrophoretic mobility assay for AuNP-cit and AuNP-MUA after Ab adsorption.


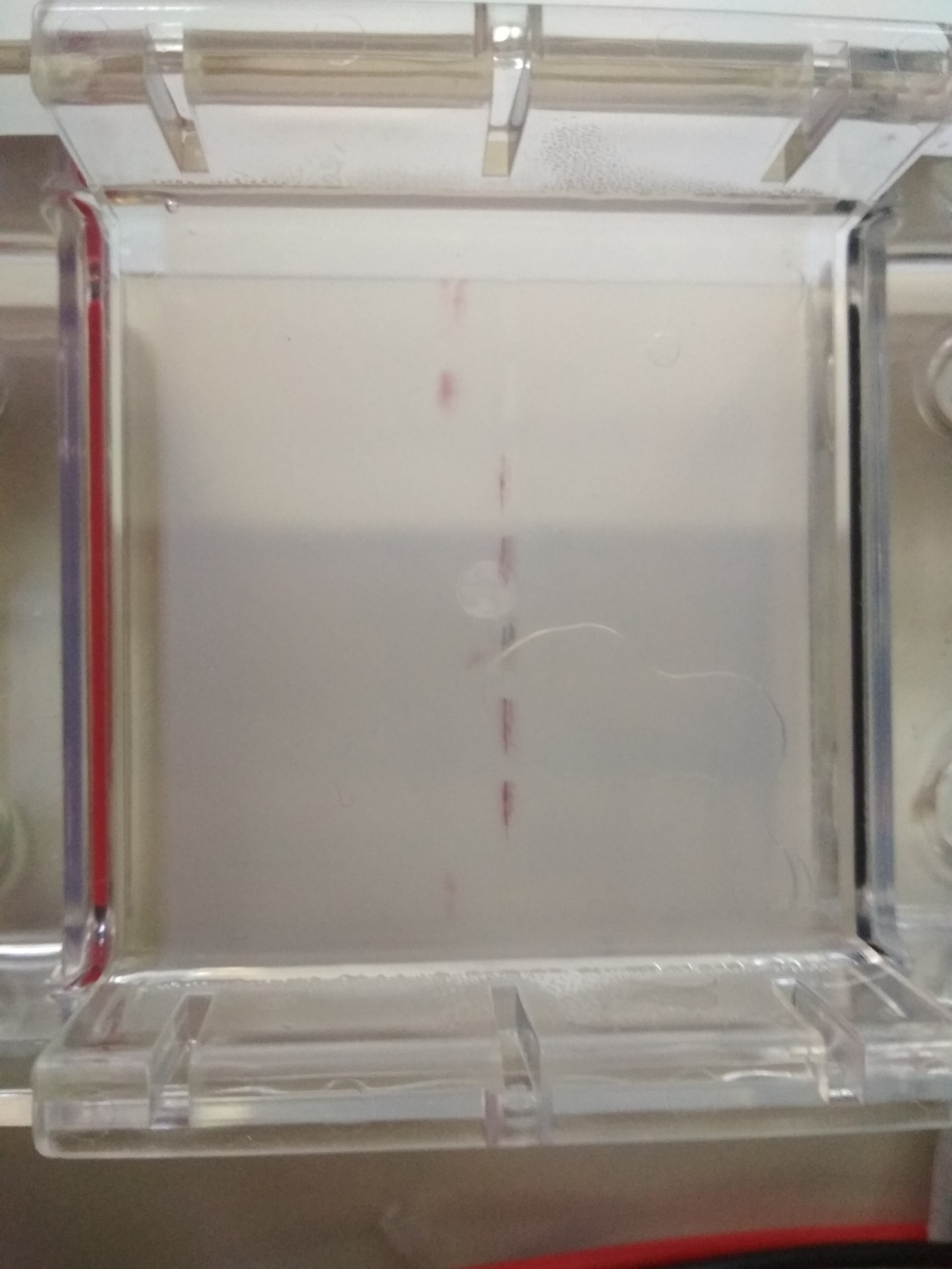


+Ab

60 min

+Ab

24 hs

-Ab

+Ab

60 min

+Ab

24 hs

-Ab

AuNP-MUA

AuNP-cit

**Figure S16. Electrophoretic mobility assay for AuNP-MUA and AuNP-cit 60 min and 24 hs after Ab adsorption (cropped from Figure S17).**


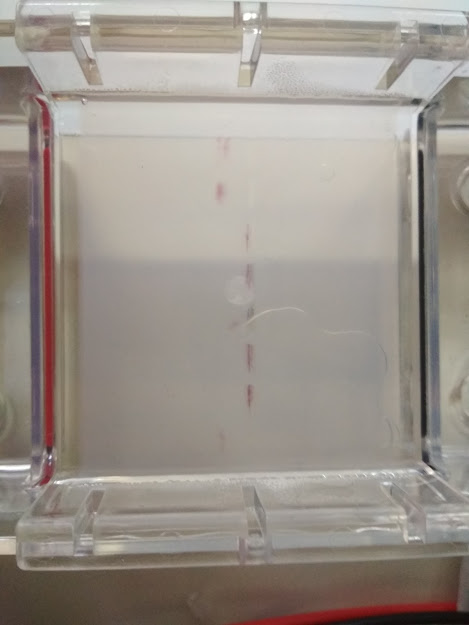


**Figure S17. Electrophoretic mobility assay for AuNP-MUA and AuNP-cit 60 min and 24 hs after Ab adsorption.** Full view.

## SM14. The effect of Ab concentration and pH in the adsorption

The effect of Ab concentration and pH in the adsorption process on AuNP-cit was negligible, as deduced from the constancy in the ratio Abs 522 nm/Abs 650 nm at 140 mM NaCl (Fig. S18). However, for AuNP-MUA-Ab, the absorbance ratio decreases with both Ab concentration and pH (Fig. S18). Above pH 8.5, less stable AuNP-MUA-Ab was formed and NaCl addition caused the formation of aggregates, as revealed by a decrease in the absorbance ratio 520nm/650nm. In this case, we argued that poorly Ab adsorption occurs. Considering that MUA is fully ionized at this pH [[15](#_ENREF_15)], and monoclonal Ab typical isoelectric points lye in the range 7.6 – 9.5 [[21](#_ENREF_21)], we believe that there is an electrostatic repulsion between Ab and AuNP-MUA that hinders the effectiveness of adsorption. This suggests that in the case of AuNP-cit, Ab adsorption removes citrate molecules, in accordance to the lower energy of attachment of this ligand to the AuNP surface.





**Figure S18. Effect of pH and Ab concentration during physical of Ab onto AuNP-cit and AuNP-MUA at a NaCl concentration of 140 mM.**

## SM15. Electrochemical evaluation





**Figure S19. Voltammetric response for the nanosystems adsorbed onto a gold screen-printed electrode of geometric area 0.126 cm^2^.** Arrow indicates the direction of the initial voltage scan at 0.050 V s^-1^.

**Table S3**. **Cyclic voltammetry evaluation of modified AuNPs.** Peak potential difference (Δ*E*_p_) and heterogeneous rate constant (*k*_o_) for the charge transfer from nanoparticle-systems modified gold screen-printed electrodes to the soluble redox probe [Fe(CN)_6_]^3-^. Gold electrode geometric area: 0.126 cm^2^.

| Electrochemical system | Δ*E*_p_ (V) | *k*_o_ (cm/s) |
| --- | --- | --- |
| Au | 0.106 ± 0.003 | 3.5 x 10^-3^ |
| Au-Ab | 0.112 ± 0.003 | 3.0 x 10^-3^ |
| Au-AuNP-cit | 0.114 ± 0.002 | 2.8 x 10^-3^ |
| Au-AuNP-cit-Ab | 0.190 ± 0.005 | 0.7 x 10^-3^ |

## SM16. Characterization of different AuNP-cit-protein preparations





**Figure S20. Stability colloidal assay (end-point) for different AuNP-cit-protein preparations in the presence or absence of 140 mM NaCl.** The error bars indicate the SD for two independent measurements.





**Figure S21. UV/Vis of AuNP-cit before and after incubation whit different proteins.** Inset: Normalized UV/Vis spectra. The arrow shows the red shift observed for all proteins assayed.

**Table S4**. **Hydrodynamic diameter for the different AuNP-cit-proteins.** Only the intensity data are shown**,** Δ*d*_h_ represents the difference between the hydrodynamic diameter of each AuNP-cit-protein assemble and the free AuNP-cit.

|  | *d*_h_ (nm) (by intensity) | Δ*d*_h_ (nm) ± SD | PI |
| --- | --- | --- | --- |
| AuNP-cit | 18 ± 1 | ----- | 0.26 ± 0.01 |
| AuNP-cit-BSA | 44 ± 1 | 26 ± 2 | 0.316 ± 0.004 |
| AuNP-cit-AG | 40 ± 3 | 22 ± 4 | 0.29 ± 0.01 |
| AuNP-cit-Strp | 305 ± 30 | 287 ± 31 | 0.28 ± 0.01 |
| AuNP-cit-Ab | 132 ± 6 | 114 ± 7 | 0.18 ± 0.02 |


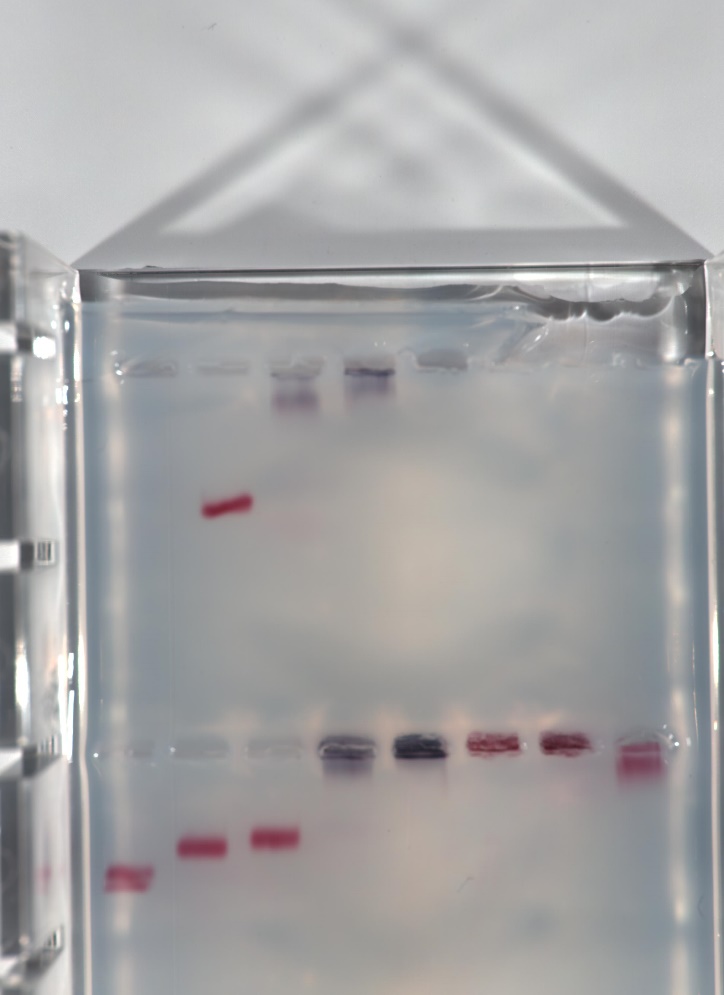


**Figure S22. Electrophoresis mobility assay**. The red rectangle shows the region cropped in Figure 5 A in main text.





**Figure S23. Size exclusion chromatography for different AuNP-cit-protein ensambles**. (A) AuNP-cit-AG, (B) AuNP-cit-BSA and free BSA (recorded at 280 nm), and (C) AuNP-cit-Strp. In all cases AuNP-MUA elution profile are included as reference. Normalized absorbances are shown for comparison.

## References

[1] J. Liu, Y. Lu, Preparation of aptamer-linked gold nanoparticle purple aggregates for colorimetric sensing of analytes, Nature protocols, 1 (2006) 246-252.

[2] W. Haiss, N.T.K. Thanh, J. Aveyard, D.G. Fernig, Determination of Size and Concentration of Gold Nanoparticles from UV−Vis Spectra, Analytical Chemistry, 79 (2007) 4215-4221.

[3] N.G. Khlebtsov, Determination of Size and Concentration of Gold Nanoparticles from Extinction Spectra, Analytical Chemistry, 80 (2008) 6620-6625.

[4] B.J. Liu, K.Q. Lin, S. Hu, X. Wang, Z.C. Lei, H.X. Lin, B. Ren, Extraction of absorption and scattering contribution of metallic nanoparticles toward rational synthesis and application, Anal Chem, 87 (2015) 1058-1065.

[5] J.R. Navarro, M.H. Werts, Resonant light scattering spectroscopy of gold, silver and gold-silver alloy nanoparticles and optical detection in microfluidic channels, Analyst, 138 (2013) 583-592.

[6] X. Liu, M. Atwater, J. Wang, Q. Huo, Extinction coefficient of gold nanoparticles with different sizes and different capping ligands, Colloids and surfaces. B, Biointerfaces, 58 (2007) 3-7.

[7] P.K. Ngumbi, S.W. Mugo, J.M. Ngaruiya, C.K. King'ondu, Multiple plasmon resonances in small-sized citrate reduced gold nanoparticles, Materials Chemistry and Physics, 233 (2019) 263-266.

[8] J. Shang, X. Gao, Nanoparticle counting: towards accurate determination of the molar concentration, Chemical Society reviews, 43 (2014) 7267-7278.

[9] E. Colangelo, J. Comenge, D. Paramelle, M. Volk, Q. Chen, R. Lévy, Characterizing Self-Assembled Monolayers on Gold Nanoparticles, Bioconjugate chemistry, 28 (2017) 11-22.

[10] D.-B. Grys, B. de Nijs, A.R. Salmon, J. Huang, W. Wang, W.-H. Chen, O.A. Scherman, J.J. Baumberg, Citrate Coordination and Bridging of Gold Nanoparticles: The Role of Gold Adatoms in AuNP Aging, ACS Nano, 14 (2020) 8689-8696.

[11] J.-W. Park, J.S. Shumaker-Parry, Structural Study of Citrate Layers on Gold Nanoparticles: Role of Intermolecular Interactions in Stabilizing Nanoparticles, Journal of the American Chemical Society, 136 (2014) 1907-1921.

[12] P. Wulandari, T. Nagahiro, K. Michioka, K. Tamada, K.-i. Ishibashi, Y. Kimura, M. Niwano, Coordination of Carboxylate on Metal Nanoparticles Characterized by Fourier Transform Infrared Spectroscopy, Chemistry Letters, 37 (2008) 888-889.

[13] J.-W. Park, Negative-Imaging of Citrate Layers on Gold Nanoparticles by Ligand-Templated Metal Deposition: Revealing Surface Heterogeneity, Particle & Particle Systems Characterization, 36 (2019) 1800329.

[14] P. Wulandari, T. Nagahiro, N. Fukada, Y. Kimura, M. Niwano, K. Tamada, Characterization of citrates on gold and silver nanoparticles, Journal of colloid and interface science, 438 (2015) 244-248.

[15] C. Lages, E. Méndez, Contact angle measurements under thermodynamic equilibrium conditions, Analytical and bioanalytical chemistry, 388 (2007) 1689-1692.

[16] M.J. Hostetler, J.J. Stokes, R.W. Murray, Infrared Spectroscopy of Three-Dimensional Self-Assembled Monolayers:  N-Alkanethiolate Monolayers on Gold Cluster Compounds, Langmuir, 12 (1996) 3604-3612.

[17] N. Sandhyarani, T. Pradeep, Current understanding of the structure, phase transitions and dynamics of self-assembled monolayers on two- and three-dimensional surfaces, International Reviews in Physical Chemistry, 22 (2003) 221-262.

[18] C. Vericat, M.E. Vela, G. Benitez, P. Carro, R.C. Salvarezza, Self-assembled monolayers of thiols and dithiols on gold: new challenges for a well-known system, Chemical Society reviews, 39 (2010) 1805-1834.

[19] J. Tang, K. Gao, Q. Ou, X. Fu, S.Q. Man, J. Guo, Y. Liu, Calculation extinction cross sections and molar attenuation coefficient of small gold nanoparticles and experimental observation of their UV-vis spectral properties, Spectrochimica acta. Part A, Molecular and biomolecular spectroscopy, 191 (2018) 513-520.

[20] L. Zhang, D. Hu, M. Salmain, B. Liedberg, S. Boujday, Direct quantification of surface coverage of antibody in IgG-Gold nanoparticles conjugates, Talanta, 204 (2019) 875-881.

[21] A. Goyon, M. Excoffier, M.-C. Janin-Bussat, B. Bobaly, S. Fekete, D. Guillarme, A. Beck, Determination of isoelectric points and relative charge variants of 23 therapeutic monoclonal antibodies, Journal of Chromatography B, 1065-1066 (2017) 119-128.
